# The *OsMYB30-OsADF7* Axis Modulates Rice Heat Acclimation Through Actin Microfilament Dynamics

**DOI:** 10.3390/plants15131976

**Published:** 2026-06-26

**Authors:** Tianying Ren, Pan Li, Zhuoqun Liu, Jingrong Wang, Tong Lu, Weiming Wang, Yichen Huang, Fuyao Wei, Lusha Ji

**Affiliations:** State Key Laboratory of Macromolecular Drugs and Large-Scale Preparation, Shandong Key Laboratory of Applied Technology for Protein and Peptide Drugs, School of Pharmaceutical Sciences and Food Engineering, Liaocheng University, Liaocheng 252059, China; dxmei_312@163.com (T.R.); lipan@lcu.edu.cn (P.L.); liuzhuoqun2004@163.com (Z.L.); keaijiang1@outlook.com (J.W.); 17793256509@163.com (T.L.); 15853912533@163.com (W.W.); huangyichen200045@163.com (Y.H.)

**Keywords:** actin depolymerization factor, heat tolerance, *Oryza sativa*, transcription factor

## Abstract

Actin cytoskeleton is a fundamental cellular structure governing stress signal transduction and cellular homeostasis in plants. While its involvement in heat stress adaptation has been documented, the transcriptional and cytoskeletal regulatory networks underlying rice thermotolerance remain poorly defined. Here, we report that the actin-depolymerizing factor *OsADF7* acts as a negative regulator of rice heat acclimation through modulating microfilament dynamics, and is transcriptionally controlled by the R2R3-MYB transcription factor *OsMYB30*. Heat stress markedly suppresses the expression of both *OsMYB30* and *OsADF7*. Functional characterization demonstrated that knockout of *Osadf* significantly enhances heat acclimation by preserving microfilament polymerization, whereas overexpression (OE) of *OsADF7* confers heat acclimation in rice seedlings. Physiological analyses including survival rate, electrolyte leakage, MDA, ROS, chlorophyll content and Fv/Fm further validated the heat acclimation phenotypes. Mechanistically, OsMYB30 directly binds to the TATCC *cis*-element in the *OsADF7* promoter and may positively regulate *OsADF7* transcription. Consequently, knockout of *Osmyb30* enhances heat tolerance, while OE of *OsMYB30* induces *OsADF7* expression and leads to heat hypersensitivity. Genetic epistasis analyses support that the *OsMYB30-OsADF7* transcriptional module may serve as a potential regulatory module involved in actin cytoskeleton-associated heat acclimation in rice. Collectively, our findings provide preliminary mechanistic clues linking MYB-related transcriptional regulation to actin cytoskeletal dynamics during rice thermotolerance responses, and provide a promising target for genetic improvement of heat-resistant rice varieties.

## 1. Introduction

Global warming poses a persistent threat to crop production worldwide, as high-temperature stress severely impairs plant growth, development, and physiological metabolism, ultimately leading to substantial yield losses and quality decline in staple crops including rice [1,2,3]. Elucidating the molecular and cellular mechanisms underlying plant thermotolerance is therefore critical for developing climate-resilient varieties and ensuring agricultural sustainability. High temperatures disrupt protein stability, membrane integrity, RNA structure, cytoskeleton organization, and enzymatic efficiency, resulting in metabolic disorders in plant cells [4,5]. Plants have evolved conserved heat-sensing systems and multi-layered regulatory networks to cope with heat stress, including calcium channels, histone sensors, and unfolded protein response sensors in the endoplasmic reticulum and cytoplasm, which collectively activate heat-responsive genes to enhance thermotolerance [6,7,8,9].

The actin cytoskeleton serves as a core regulator of cell morphology and intracellular signal transduction, and microfilaments are more sensitive to heat stress than microtubules [10,11]. Microtubules and microfilaments undergo dynamic disassembly and reassembly in response to heat, and the kinetics of cytoskeleton rearrangement vary with temperature and cell type [12,13]. As highly conserved microfilament-binding proteins, actin-depolymerizing factors (ADFs) regulate actin dynamics by severing or depolymerizing F-actin, and are widely involved in plant development, stomatal movement, and abiotic stress responses [14,15,16]. ADF family members participate in drought, salt, heat, and cold stress responses, and overexpression (OE) of *AtADF3* or *OsADF3* improves plant tolerance to multiple stresses [17,18,19]. However, the upstream transcriptional regulatory mechanism of *ADF* genes during heat acclimation in rice remains largely unclear.

MYB transcription factors, especially the R2R3-MYB subfamily, play vital roles in plant development, metabolism, and stress responses [20,21,22]. *MYB30* is a well-characterized R2R3-MYB regulator involved in various stress responses, but its connection with actin cytoskeleton regulators in heat tolerance has not been reported [23]. Rice is a staple crop worldwide, and dissecting its heat adaptation mechanisms is of great importance for production [24]. Current studies on rice thermotolerance mainly focus on heat-induced transcription factors and heat shock proteins (HSPs), while the regulatory network of microfilament cytoskeleton remains poorly understood. We hypothesized that *OsMYB30* transcriptionally regulates *OsADF7* to modulate actin dynamics and heat tolerance in rice. The aims of this study were: (1) to characterize the function of *OsADF7* in heat stress; (2) to verify that OsMYB30 directly binds the *OsADF7* promoter; (3) to reveal the *OsMYB30–OsADF7* regulatory module in heat acclimation; and (4) to provide a theoretical basis for heat-resistant rice breeding.

## 2. Results

### 2.1. Bioinformatics Analysis of Rice Microfilament Depolymerization Factor OsADF7

**Figure 1:**
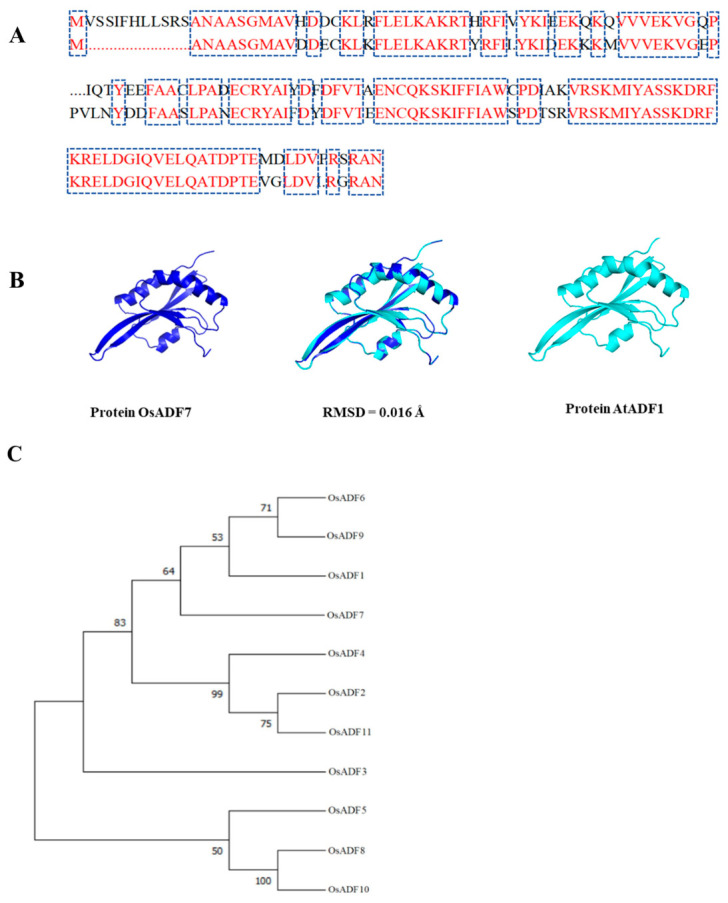
Bioinformatics analysis of rice microfilament depolymerization factor OsADF7. (**A**) Protein sequence identity between OsADF7 and Arabidopsis AtADF1 was 79.90%. (**B**) Three-dimensional structural superposition of OsADF7 and Arabidopsis AtADF1. (**C**) Phylogenetic tree of the rice ADF family constructed using Molecular Evolutionary Genetics Analysis (MEGA) software.

To identify the ADF family member involved in heat stress response in rice, the protein sequence of *Arabidopsis thaliana AtADF1* was retrieved from the TAIR database and used as a query for basic local alignment search tool (BLAST version 2.10.0) searches against the National Rice Data Center and NCBI databases. A homologous sequence (accession No. NM_003074.8.001420040.1) was obtained and designated as *OsADF7*. Sequence analysis showed that the full-length genomic sequence of *OsADF7* was 779 bp, containing a 417 bp open reading frame encoding 139 amino acids, with a predicted molecular weight of 15.86 kDa and an isoelectric point (pI) of 5.22 (Figure 1A). Multiple sequence alignment revealed that OsADF7 shared 79.90% identity with AtADF1 and contained a conserved ADF domain (Figure 1A). Three-dimensional structure homology modeling indicated that OsADF7 exhibited a highly similar folding pattern to AtADF1 (PDB: 1F7S) with a low root-mean-square deviation (RMSD) value (Figure 1B). Phylogenetic analysis of the 11 identified rice ADF family members showed that OsADF7 formed an independent clade, suggesting its functional specificity (Figure 1C).

### 2.2. Expression Profiling of the OsADF Gene Family Under Heat Stress

**Figure 2:**
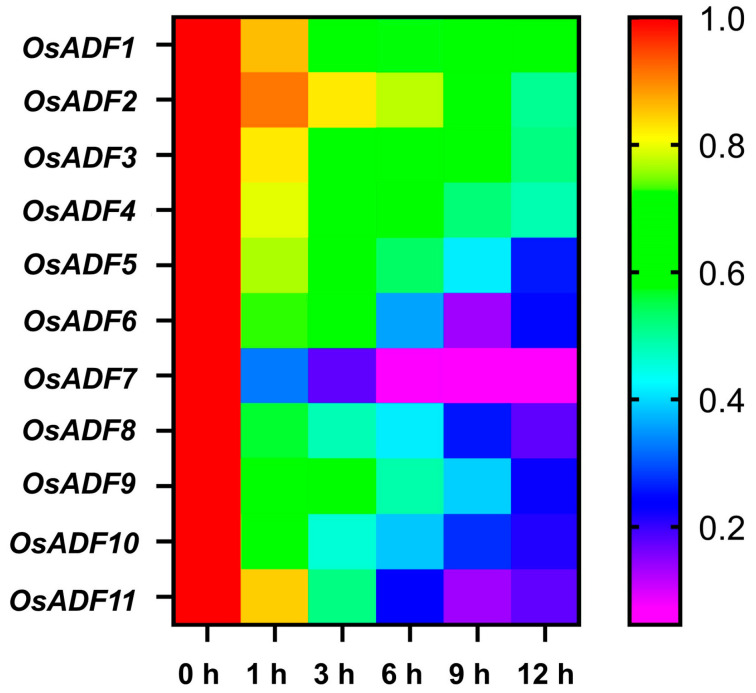
Expression profiles of the OsADF gene family in rice seedlings under gradual heat stress at 37 °C. Fifteen-day-old seedlings precultured at 22 °C were exposed to 37 °C at 0, 1, 3, 6, 9, and 12 h. Rows represent individual *OsADF* genes: 1, *OsADF1*; 2, *OsADF2*; 3, *OsADF3*; 4, *OsADF4*; 5, *OsADF5*; 6, *OsADF6*; 7, *OsADF7*; 8, *OsADF8*; 9, *OsADF9*; 10, *OsADF10*; 11, *OsADF11*. The color gradient from red to purple indicates relative transcript levels from highest (1.0) to lowest (0.0), as quantified by qRT-PCR. Note: Data are presented as means (n = 3 biological replicates; each with three technical replicates).

To identify *OsADF* members involved in heat acclimation, transcript levels of 11 OsADF family genes were quantified by real-time polymerase chain reaction (qRT-PCR) in WT rice seedlings exposed to 37 °C acute heat stress for 0, 1, 3, 6, 9, and 12 h. Most *OsADF* members showed negligible expression changes, whereas *OsADF7* was strongly and progressively downregulated under acute heat stress (Figure 2). To validate whether this transcriptional response is conserved under agronomically relevant chronic moderate heat, *OsADF7* transcript levels were further detected at 28 °C for 0, 2, 4, and 6 d. *OsADF7* was also significantly downregulated in a time-dependent manner at 28 °C, exhibiting identical repression kinetics to those at 37 °C (Figure S1). This conserved transcriptional repression of *OsADF7* under both acute extreme heat and chronic moderate heat demonstrates that the downregulation of *OsADF7* is a key conserved molecular signature underlying rice heat acclimation. The rapid transcriptional response at 37 °C initiates the heat acclimation program, while the sustained repression at 28 °C translates this molecular signal into stable cytoskeletal and phenotypic outcomes. Together, these dynamic regulatory processes establish a direct mechanistic link between acute transcriptional responses to sustained thermotolerance under field-like conditions.

### 2.3. Functional Characterization of OsADF7 in Rice Heat Stress Response Through Modulating Microfilament Polymerization

#### 2.3.1. Validation of *OsADF7* Knockout Lines and Involvement of Nonsense-Mediated mRNA Decay (NMD)

To functionally dissect *OsADF7*, two independent homozygous knockout lines (*ko1* and *ko2*) were generated via clustered regularly interspaced short palindromic repeats/CRISPR-associated protein 9 (CRISPR/Cas9)-mediated genome editing. The gene structure, CRISPR target site and mutation details of *OsADF7* are illustrated in Figure S2A. Sanger sequencing confirmed that *ko1* carried a 13-bp deletion causing a frameshift and a premature termination codon (PTC), while *ko2* harbored a 3-bp insertion that disrupted the conserved ADF domain (Figure S2B).

**Figure 3:**
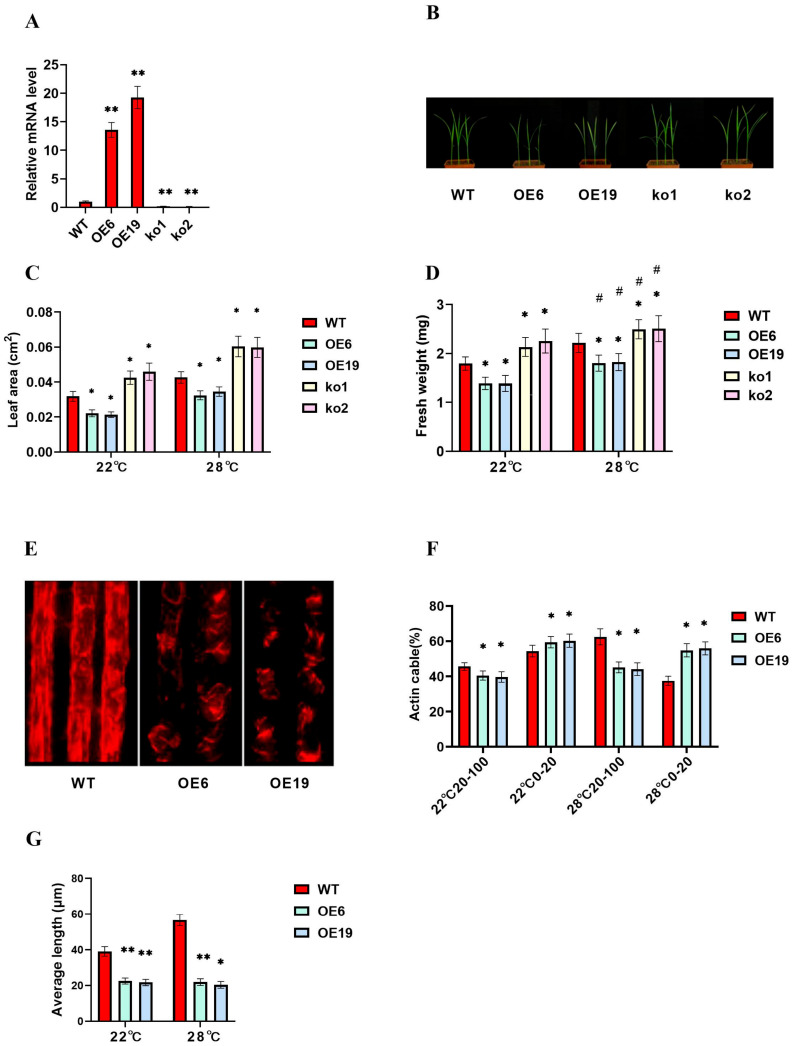
Phenotypic and cytoskeletal characterization of *OsADF7* transgenic rice seedlings under high-temperature stress. (**A**) qRT-PCR confirmation of *OsADF7* overexpression (*OE6*, *OE19*) and knockout (*ko1*, *ko2*) lines. (**B**) Representative phenotypes of WT, *OE*, and *ko* seedlings following 4 days exposure to 28 °C (moderate heat stress). (**C**) Quantification of leaf area in WT, *OE*, and *ko* lines under control (22 °C) and heat stress (28 °C) conditions. (**D**) Proportion of depolymerized (low-fluorescence) actin filaments in leaf pavement cells, determined by K-means clustering of fluorescence intensity distribution (see Figure S3). # *p* < 0.05 compared with 22 °C at same conditions. (**E**) Average actin filaments length in leaf pavement cells. (**F**) Quantification of the proportion of high-fluorescence (20–100, polymerized) and low-fluorescence (0–20, depolymerized) actin cables in WT, *OE6* and *OE19* lines under control (22 °C) and moderate high-temperature stress (28 °C) conditions. (**G**) Quantification of average actin filament length in WT, *OE6* and *OE19* lines under control (22 °C) and moderate high-temperature stress (28 °C) conditions. Note: Values are means ± standard error (SE). * *p* < 0.05, ** *p* < 0.01 compared with WT at same condition. n = 3 biological replicates, each with three technical replicates per biological sample. At least 1000 microfilaments were quantified per genotype. Statistical significance was determined by two-way ANOVA followed by Bonferroni’s post hoc test.

qRT-PCR analysis revealed that mature *OsADF7* transcript levels were significantly reduced by 78% in *ko1* and 65% in *ko2* relative to WT plants (Figure 3A). Conversely, *OsADF7* pre-mRNA levels did not differ significantly between the knockout lines and WT (*p* > 0.05), demonstrating unaltered transcriptional initiation. Concurrently, transcript levels of the core NMD pathway genes *OsUPF1*, *OsUPF2* and *OsUPF3* were markedly upregulated in both ko1 and ko2 (*p* < 0.05; Figure S2C).

Together, these results demonstrate that the reduced mature *OsADF7* mRNA in the knockout lines arises from NMD-mediated degradation of aberrant transcripts containing PTC or disrupted functional domains, rather than impaired transcription.

#### 2.3.2. *OsADF7* Negatively Regulates Rice Heat Acclimation Under Moderate High Temperature (28 °C)

To determine the biological function of *OsADF7* during heat stress, homozygous OE lines (*OE6*, *OE19*) and knockout lines (*ko1*, *ko2*) were obtained via Agrobacterium-mediated transformation and three generations of resistance screening (Figure 3A). Seedlings of WT, *OE*, and *ko* lines were cultured at 22 °C for 3 days, then exposed to moderate high-temperature stress (28 °C) for 4 days. Growth performance was evaluated by measuring leaf area and fresh weight.

Compared with 22 °C control, 28 °C chronic heat stress significantly increased leaf area and fresh weight across all genotypes (*p* < 0.05), consistent with adaptive growth promotion under moderate heat (Figure 3B–D) [13]. Under moderate high temperature (28 °C) for heat acclimation, *OsADF7-OE* lines displayed impaired growth, whereas *Osadf7-ko* lines showed enhanced growth relative to WT (*p* < 0.05).

To reliably evaluate heat acclimation capacity, we further detected six canonical physiological indicators: survival rate after recovery from heat stress, electrolyte leakage (EL), malondialdehyde (MDA), reactive oxygen species (ROS) accumulation, total chlorophyll content and maximum photochemical efficiency of PSII (Fv/Fm) (Table S1). Compared with WT, *Osadf7-ko* lines showed significantly higher survival rate (92.3 ± 3.1%), higher chlorophyll content, higher Fv/Fm value, lower EL, lower MDA and lower ROS accumulation; in contrast, *OsADF7-OE* lines exhibited lower survival rate (51.7 ± 4.2%), lower chlorophyll content, lower Fv/Fm value, higher EL, higher MDA and higher ROS levels (*p* < 0.05). These physiological data consistently support that *OsADF7* acts as a negative regulator of rice heat acclimation.

#### 2.3.3. *OsADF7* Modulates Microfilament Polymerization in Rice Leaf Pavement Cells Under High Temperature

Actin microfilaments are core cytoskeletal components that govern plant growth, cell morphogenesis, signal transduction, and stress adaptation, including thermotolerance responses. To define the role of *OsADF7* in regulating actin cytoskeletal organization under high-temperature stress, we quantitatively analyzed actin bundling intensity and filament length in leaf pavement cells of WT and *OsADF7-OE* lines (*OE6*, *OE19*). Fluorescence intensity of all microfilaments was quantified in full scale, and a bimodal distribution was determined by histogram plotting (Figure S3); microfilaments were objectively classified into low-fluorescence (depolymerized) and high-fluorescence (polymerized) populations via K-means clustering, eliminating arbitrary threshold partitioning.

Under normal temperature (22 °C), the proportion of low-fluorescence (depolymerized) actin filaments was significantly higher, while high-fluorescence (polymerized) bundles were markedly lower in *OE6* and *OE19* relative to WT (Figure 3D). These observations indicate that *OsADF7 OE* attenuates actin filament bundling under basal conditions. Following exposure to high temperature (28 °C), all genotypes exhibited a general increase in low-fluorescence filaments and a reduction in high-fluorescence bundles compared with the control condition (Figure 3D). Quantification of average actin filament length revealed that high-temperature treatment increased filament length in WT, *OE6*, and *OE19*. Notably, under high-temperature stress, average actin filament length remained significantly lower in *OE6* and *OE19* than in WT (Figure 3E–G).

Together, these findings demonstrate that *OsADF7 OE* strongly suppresses actin microfilament polymerization and bundling in rice leaf pavement cells under high-temperature stress, supporting a regulatory role for *OsADF7* in cytoskeletal remodeling during thermal adaptation.

### 2.4. Effect of OsADF7 on the Expression of Heat-Responsive Genes Under High Temperature

HSPs are central mediators of plant thermotolerance, and accumulating evidence indicates that actin cytoskeleton integrity closely modulates HSP-mediated heat stress responses in eukaryotes. To explore the molecular link between *OsADF7*-dependent actin regulation and downstream heat signaling, we quantified transcript levels of core *HSP* genes in WT, *OsADF7-OE* (*OE6*, *OE19)* and knockout (*ko1*, *ko2*) lines before and after 37 °C heat treatment for 6 h.

**Figure 4:**
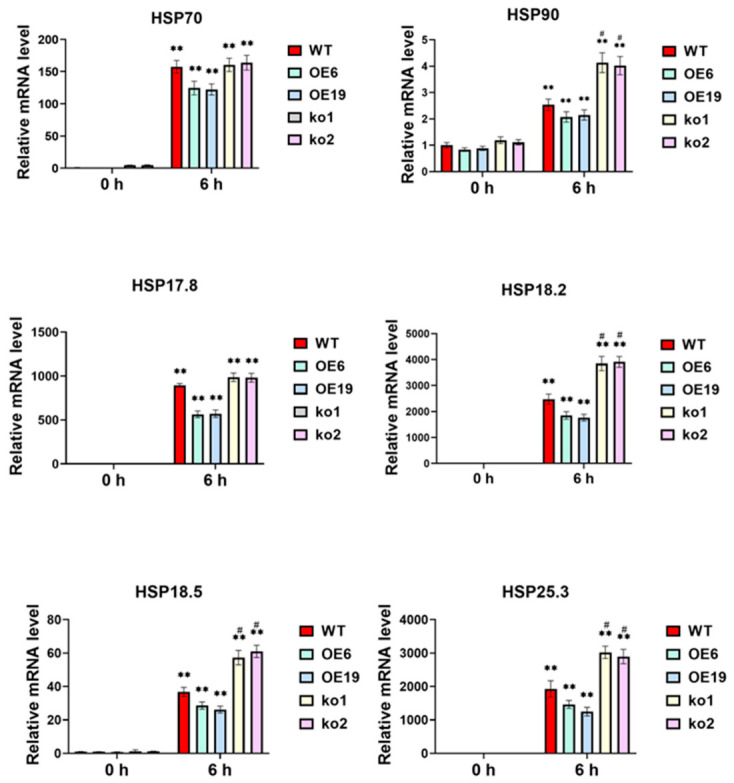
Expression analysis of heat-responsive genes in *OsADF7* transgenic rice under heat stress by qRT-PCR. Note: Values are means ± SE from three biological replicates (each with three technical replicates). ** *p* < 0.01 vs. 0 h; # *p* < 0.05 vs. wild-type (WT). Two-way ANOVA followed by Bonferroni’s post hoc test was used for statistical comparisons.

Under control conditions (0 h), no significant differences in *HSP* gene expression were detected among all genotypes (Figure 4). After exposure to 37 °C heat stress for 6 h, the expression of *OsHSP70*, *OsHSP90*, *OsHSP17.8*, *OsHSP18.2*, *OsHSP18.5* and *OsHSP25.3* was significantly upregulated in all genotypes compared with the 0 h control.

Notably, genotype-specific expression patterns emerged: transcript levels of *OsHSP18.5* and *OsHSP25.3* were significantly lower in WT and *OsADF7-OE* lines than in *Osadf7-ko* lines. Conversely, *OsHSP18.5* and *OsHSP25.3* were highly upregulated in *Osadf7-ko1* and *Osadf7-ko2* plants. These results reveal that *OsADF7* negatively regulates the induction of key *HSP* genes under heat stress, supporting a mechanistic connection between actin cytoskeleton dynamics and transcriptional activation of the heat stress response pathway.

### 2.5. Effect of High Temperature on OsMYB30 Expression

**Figure 5:**
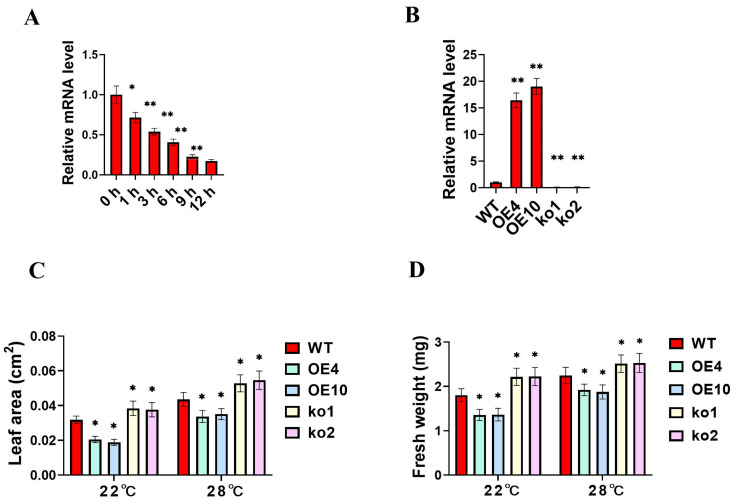
Expression profiling and phenotypic characterization of *OsMYB30* under high-temperature stress. (**A**) qRT-PCR analysis of *OsMYB30* expression in wild-type (*WT*) rice (cv. Nipponbare) under heat stress. (**B**) Expression validation of *OsMYB30* in transgenic lines by qRT-PCR. (**C**) Leaf area of *OsMYB30* transgenic seedlings under high-temperature stress. (**D**) Fresh weight of *OsMYB30* transgenic seedlings under high-temperature stress. Note: Values are means ± SE. * *p* < 0.05, ** *p* < 0.01 vs. 0 h or WT. Statistical significance was determined by two-way ANOVA followed by Bonferroni’s post hoc test.

*OsMYB30* acts as the upstream transcriptional regulator of *OsADF7* in the heat acclimation pathway. qRT-PCR analysis showed that *OsMYB30* was significantly downregulated in a time-dependent manner under both 37 °C acute heat stress and 28 °C chronic moderate heat stress (Figure 5A and Figure S4), mirroring the expression pattern of *OsADF7*. This coordinated repression of the *OsMYB30-OsADF7* axis under both temperature regimes indicate that this regulatory module is a conserved core switch for rice heat acclimation. The rapid downregulation at 37 °C serves as the molecular trigger for heat acclimation, while the sustained repression at 28 °C drives the cytoskeletal remodeling and growth adaptation required for long-term thermotolerance, establishing a direct mechanistic link between the acute transcriptional response and the chronic phenotypic and cytoskeletal outcomes observed under agronomically relevant conditions.

To establish the functional link between *OsMYB30* and *OsADF7*, *OsMYB30-OE* (*OE4*, *OE10*) and knockout (*ko1*, *ko2*) lines were generated via Agrobacterium-mediated transformation and validated to homozygosity after three generations of screening (Figure 5B). Phenotypic analysis was conducted using WT, *OE*, and *ko* seedlings grown at 22 °C for 3 d followed by exposure to 28 °C for 4 d. Leaf area and fresh weight were measured to evaluate growth performance (Figure 5C,D).

Compared with normal conditions (22 °C), all genotypes exhibited significantly increased leaf area and fresh weight under moderate high temperature (28 °C) for heat acclimation. Notably, *Osmyb30-ko1* and *Osmyb30-ko2* displayed significantly larger leaf area and higher fresh weight than WT, whereas *OsMYB30-OE4* and *OsMYB30-OE10* showed markedly reduced growth relative to *WT* under identical moderate high-temperature condition.

### 2.6. OsMYB30 Binds to the OsADF7 Promoter In Vitro and In Vivo

**Figure 6:**
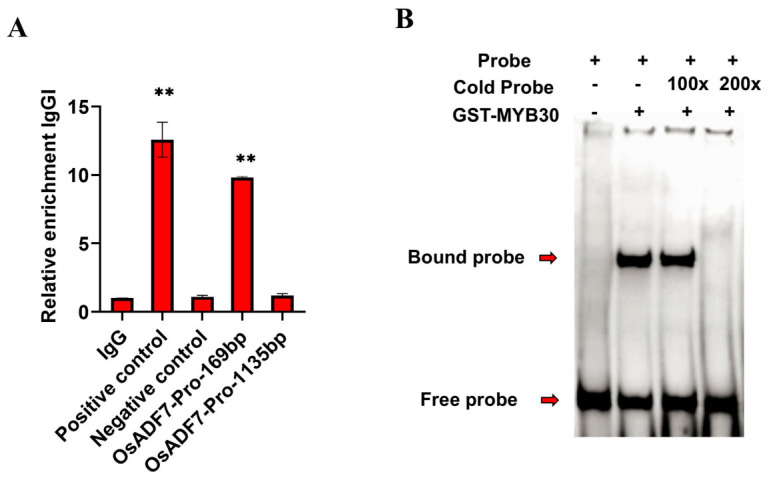
OsMYB30 directly binds to the *OsADF7* promoter in vitro and in vivo. (**A**) ChIP-qPCR validation of *OsMYB30* occupancy on the OsADF7 promoter in planta. ChIP was performed with anti-GFP antibody using 14-day-old OsMYB30-GFP seedlings; IgG was negative control and Input was normalization reference. *OsHSP25.3* promoter (known OsMYB30-binding site) was positive control, and intergenic region without TATCC motif was negative control. Pro-169 bp: *OsADF7* promoter fragment with TATCC; Pro-1135 bp: fragment without TATCC. Data are means ± SE (n = 3). ** *p* < 0.01 vs. IgG (one-way ANOVA with Tukey’s test). Note: Values are means ± SE. ** *p* < 0.01 compared with Mock. n = 3 biological replicates with three technical replicates per sample. Statistical significance was determined by one-way ANOVA followed by Tukey’s post hoc test. (**B**) Electrophoretic mobility shift assay (EMSA) verifying OsMYB30 binding to the *OsADF7* promoter in vitro.

Previous studies have documented that OsMYB30 recognizes and binds to conserved cis-elements including AACAAAC, CAGTTG, TATCC, and TTTGGTT. Promoter sequence analysis and schematic diagram (Figure S5) identified two canonical OsMYB30-binding TATCC motifs located at −169 bp and −1135 bp upstream of the *OsADF7* transcription start site (TSS); this promoter schematic clearly delineates the target regions for subsequent chromatin immunoprecipitation–quantitative polymerase chain reaction (ChIP-qPCR) and electrophoretic mobility shift assay (EMSA) assays. To verify the in vivo direct binding of OsMYB30 to the *OsADF7* promoter, ChIP-qPCR was performed. The results showed significant enrichment of OsMYB30 at the −169 bp region of the *OsADF7* promoter, whereas no obvious enrichment was detected at the −1135 bp region (Figure 6A). These data indicate that OsMYB30 specifically associates with the −169 bp region of the *OsADF7* promoter in planta.

For in vitro validation, recombinant OsMYB30 glutathione S-transferase (GST) fusion protein was purified and used in EMSA. Biotin-labeled (hot) and unlabeled (cold) probes corresponding to the −169 bp fragment of the *OsADF7* promoter were synthesized. The intensity incubation of OsMYB30-GST with the hot probe yielded a distinct shifted band, indicating direct protein-DNA binding. The intensity of the shifted band was attenuated by the addition of 100-fold and 200-fold excess cold probe, respectively (Figure 6B). Together, these results indicate that OsMYB30 directly and specifically binds to the TATCC motif within the −169 bp region of the *OsADF7* promoter both in vitro and in vivo.

### 2.7. OsMYB30 May Positively Regulate OsADF7 Transcription to Negatively Regulate Rice Heat Acclimation

**Figure 7:**
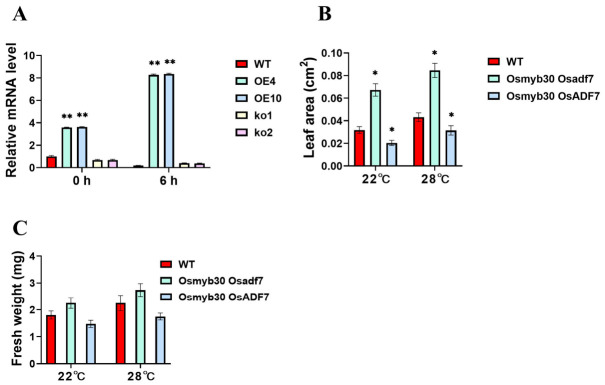
OsMYB30 modulates *OsADF7* expression and heat tolerance in rice under high-temperature stress. (**A**) qRT-PCR analysis of *OsADF7* transcript levels in *OsMYB30* transgenic lines under heat stress. * *p* < 0.05, ** *p* < 0.01 compared with wild-type (WT); n = 3. (**B**) Leaf area and (**C**) fresh weight of *WT*, *osmyb30 osadf7* double mutant, and *osmyb30 OsADF7-OE* seedlings at 22 °C and 28 °C; 1, *WT*; 2, *osmyb30 osadf7*; 3, *osmyb30 OsADF7-OE*.

Both *OsADF7* and *OsMYB30* act as negative regulators of thermotolerance in rice. Given that OsMYB30 directly binds to the *OsADF7* promoter, we validated their transcriptional regulatory relationship via qRT-PCR in WT, *OsMYB30-OE* (*OsMYB30-OE*; *OE4*, *OE10*), and *OsMYB30*-knockout (*Osmyb30-ko*; *ko1*, *ko2*) lines under control (22 °C) and heat-stress (37 °C for 6 h) conditions (Figure 7A). *OsADF7* transcript abundance was significantly elevated in *OsMYB30-OE* lines but reduced in *Osmyb30-ko* lines under both conditions, suggesting that *OsMYB30* may positively regulate *OsADF7* transcription.

To genetically define the linear epistatic pathway (*OsMYB30* upstream of *OsADF7*), we generated two combinatorial genetic lines via sexual crossing: (1) *osmyb30 osadf7* double-knockout (*DKO*; *Osmyb30-ko1* × *Osadf7-ko1*); (2) *Osmyb30-ko OsADF7-OE* (*Osmyb30-ko1* × *OsADF7-OE19*). Phenotypic and physiological analysis was performed under control (22 °C) and moderate high temperature (28 °C for 4 d) for heat acclimation conditions, with leaf area, fresh weight and six canonical heat acclimation indicators (survival rate, electrolyte leakage, MDA, ROS, chlorophyll content, Fv/Fm) quantified as comprehensive evaluation indexes (Figure 7B,C; Table S1).

Genetic epistasis analyses uncovered three core findings: (1) The *Osadf7-ko* mutation completely rescued the heat-hypersensitive phenotype of *OsMYB30-OE* plants, with *OsMYB30-OE Osadf7-ko* lines displaying thermotolerance equivalent to the *Osadf7-ko* single mutant. (2) The *Osmyb30-ko* mutation failed to rescue the heat-hypersensitive phenotype of *OsADF7-OE* plants, with *Osmyb30-ko OsADF7-OE* lines retaining the heat-sensitive phenotype of the *OsADF7-OE* single mutant. (3) The *Osmyb30 Osadf7* double-knockout (DKO) line exhibited significantly higher thermotolerance than both single mutants, and this enhanced tolerance was attributed to improved cellular homeostasis and cytoskeletal stability rather than de-repression of *OsADF7* transcription (*OsADF7* transcript levels were comparable between the *DKO* and single mutants).

These genetic epistasis analyses preliminarily suggest that *OsMYB30* may function upstream of *OsADF7* in the negative regulation of rice heat acclimation, with heat tolerance of the *Osmyb30 osadf7 DKO* line driven by enhanced cellular robustness rather than altered *OsADF7* expression.

## 3. Discussion

### 3.1. Functional Validation of Osadf7 Knockout Lines and the Role of the Nonsense-Mediated mRNA Decay (NMD) Pathway

Robust validation of gene-edited lines is essential for reliable functional characterization. In this study, two independent *Osadf7* knockout lines (*ko1* and *ko2*) were generated via CRISPR/Cas9-mediated genome editing. Line *ko1* carries a 12-bp deletion that causes a frameshift and a PTC, whereas *ko2* contains a 3-bp insertion that disrupts the conserved ADF functional domain. Strikingly, mature *OsADF7* transcript levels were drastically reduced in both knockout lines, a phenotype indicative of activated NMD, a highly conserved post-transcriptional quality-control mechanism in eukaryotes.

CRISPR/Cas9-induced lesions often generate aberrant transcripts harboring PTCs or disrupted coding regions. Such defective mRNAs are selectively recognized and degraded by the NMD pathway to prevent the synthesis of truncated, potentially deleterious proteins [25,26]. Our molecular data support this model: pre-mRNA levels of *OsADF7* were unchanged in *ko1* and *ko2* compared with WT plants, excluding transcriptional repression as the cause of reduced mature transcripts. Concurrently, the core NMD marker genes *OsUPF1*, *OsUPF2*, and *OsUPF3* were significantly upregulated in the knockout lines, directly confirming NMD pathway activation. These results demonstrate that diminished mature *OsADF7* transcripts arise from NMD-mediated degradation of aberrant mRNAs, not from impaired transcription.

NMD-triggered transcript clearance is a widespread phenomenon in plant genome-editing systems. For example, NMD activation has been documented in *Arabidopsis ADF* knockout lines, where frameshift mutations elicited selective degradation of target transcripts [27]. Parallel findings were reported in rice *OsHSP* knockout mutants, in which NMD ensured the removal of non-functional transcripts [17]. In the present study, NMD activation in *OsADF7* knockout lines not only validates the high specificity and efficiency of CRISPR/Cas9 editing but also ensures the functional null status of *OsADF7*. When integrated with phenotypic and cytoskeletal analyses, these data support that *OsADF7* acts as a negative regulator of heat acclimation in rice.

### 3.2. OsADF7 Negatively Regulates Rice Heat Tolerance by Modulating Actin Microfilament Polymerization

The dual-temperature paradigm, which uses 37 °C acute heat stress for transcriptional profiling and 28 °C chronic moderate stress for phenotypic and cytoskeletal assays, faithfully recapitulates the spatiotemporal heat stress dynamics in rice paddy ecosystems and establishes a rigorous mechanistic framework for dissecting heat acclimation. Under field conditions, rice initially encounters transient extreme heatwaves that rapidly initiate genome-wide transcriptional reprogramming; this acute molecular response is followed by prolonged mild elevated temperature throughout the growing season, which stabilizes adaptive growth and cytoskeletal remodeling to sustain long-term thermotolerance.

Our data demonstrate that the rapid transcriptional repression of *OsADF7* at 37 °C is not a transient stress reaction, but a priming event that may contribute to the heat acclimation program. This regulatory pattern is stably maintained at 28 °C, resulting in attenuated actin depolymerization, preserved cytoskeletal integrity, and enhanced heat tolerance. The strict coherence between *OsADF7* expression and phenotypic outputs at 28 °C validates that the acute transcriptional response at 37 °C serves as the molecular basis for chronic adaptive phenotypes, revealing a canonical time–intensity coupled regulatory mechanism in plant heat acclimation: transient extreme heat activates the core transcriptional module, whereas sustained moderate heat consolidates regulatory output to confer stable thermotolerance.

ADFs serve as central regulators of actin cytoskeleton dynamics, which are indispensable for plant growth, development, and environmental stress adaptation. Genome-wide studies have established that the ADF gene family is widely implicated in abiotic stress responses, with numerous members in Medicago sativa strongly induced by salt and drought stress, underscoring their evolutionary conserved functions in stress tolerance. The present work significantly advances this framework by demonstrating that *OsADF7*, a specific member of the rice ADF family, functions as a key negative regulator of heat stress responses.

Phenotypic analysis under 28 °C chronic moderate heat stress showed that *Osadf7-ko* lines exhibited enhanced growth, while *OsADF7-OE* lines showed growth inhibition relative to WT. Critically, *OsADF7* was significantly downregulated at 28 °C (moderate high temperature for heat acclimation), and this downregulation was largely attributed to reduced actin depolymerization and preserved cytoskeletal integrity. Phenotypic and physiological indicators (survival rate, EL, MDA, ROS, chlorophyll, Fv/Fm) jointly indicated the heat acclimation phenotype. For *japonica rice* cv. Nipponbare, the optimal growth temperature is 22–25 °C, and 28 °C represents a moderate chronic heat stress that induces adaptive cytoskeletal and physiological responses without severe cellular damage [28,29,30]. This direct correspondence between *OsADF7* expression and phenotype at 28 °C confirms that the observed thermotolerance is driven by consistent molecular regulation under the same stress condition.

As fundamental cytoskeletal elements, actin microfilaments maintain cellular structural integrity and transduce stress signals under adverse environments. Using objective fluorescence distribution and K-means clustering (Figure S3), our quantitative analysis of actin organization in leaf pavement cells provides unbiased mechanistic insight into *OsADF7* function. Under both control (22 °C) and heat-stress (28 °C) conditions, *OsADF7 OE* lines exhibited a higher proportion of low-fluorescence (depolymerized) actin filaments and shorter average filament length relative to *WT*. These observations demonstrate that *OsADF7* negatively regulates actin polymerization and bundle formation.

Notably, high-temperature stress alone triggered pronounced actin remodeling in *WT* plants, characterized by an increased proportion of weakly fluorescent filaments and longer average filament length relative to control conditions. This indicates that dynamic actin reorganization is an intrinsic adaptive response of rice to heat stress. However, *OsADF7-OE* exacerbated the heat-induced reduction in actin polymerization, likely compromising cellular structural stability and stress signaling, thereby reducing heat tolerance. In contrast, loss of *OsADF7* function preserves actin polymerization capacity under heat stress, reinforcing cytoskeletal stability and enhancing plant thermotolerance.

### 3.3. Implications and Future Directions

This work identifies a previously uncharacterized function of *OsADF7* in governing rice heat tolerance via the dynamic regulation of actin microfilament polymerization, thereby advancing the mechanistic understanding of plant thermotolerance. The negative regulatory role of *OsADF7* highlights a promising target for molecular breeding: targeted repression of *OsADF7* through CRISPR/Cas9-mediated gene editing or RNA interference represents a feasible strategy to enhance heat resilience in rice cultivars, which is of critical importance given global climate warming and the increasing incidence of extreme high-temperature events.

In addition, the activation of the NMD pathway in *Osadf7-ko* lines underscores the necessity of integrating post-transcriptional regulation into the validation of genome-edited materials. The combinatorial approach employed herein, which includes genotyping, quantification of pre-mRNA and mature mRNA, and expression analysis of core NMD marker genes, establishes a rigorous and systematic framework for verifying knockout efficiency. This strategy can be broadly adopted in functional genomic studies across plant species.

Despite these advances, multiple key questions remain to be explored in future investigations. First, the direct biochemical interaction between *OsADF7* and actin filaments under heat stress should be further defined using in vitro reconstitution systems, including actin depolymerization and severing assays. Second, the upstream transcriptional and signaling regulators that modulate *OsADF7* expression under heat stress remain to be identified; elucidating these components will help reconstruct the complete regulatory network governing actin cytoskeleton-mediated heat adaptation. Third, the potential crosstalk between *OsADF7*-dependent actin remodeling and other core stress pathways requires systematic dissection to achieve an integrated understanding of rice thermotolerance. These core pathways include hormone signaling, reactive oxygen species (ROS) homeostasis, and protein quality control.

## 4. Materials and Methods

### 4.1. Bioinformatics

Nucleotide and protein sequences of OsADF family genes were retrieved from the National Rice Data Center (www.ricedata.com, accessed on 21 April 2025) and the National Center for Biotechnology Information (NCBI, https://www.ncbi.nlm.nih.gov/, accessed on 21 April 2025). The protein sequence of *Arabidopsis thaliana* AtADF1 was obtained from The Arabidopsis Information Resource (TAIR, https://www.arabidopsis.org/, accessed on 21 April 2025). Multiple sequence alignment between *OsADF7* and *AtADF1* was performed using CLUSTALW (https://www.genome.jp/tools-bin/clustalw, accessed on 21 April 2025), and the alignment result was visualized with ESPript (http://espript.ibcp.fr/ESPript/cgi-bin/ESPript.cgi, accessed on 21 April 2025). The theoretical molecular weight and pI of OsADF7 protein were computed using the ProtParam tool (https://web.expasy.org/protparam/, accessed on 23 April 2025). Three-dimensional structural homology modeling of OsADF7 was conducted using the SWISS-MODEL server (https://swissmodel.expasy.org/interactive, accessed on 23 April 2025). Phylogenetic analysis of OsADF family members and AtADF1 was implemented using MEGA 7.0 software. The phylogenetic tree was constructed using the neighbor-joining (NJ) method with 1000 bootstrap replicates to evaluate branch reliability.

### 4.2. Generation of Transgenic Rice Lines for OsADF7 and OsMYB30

Transgenic rice lines (*OE* and *ko*) of *OsADF7* and *OsMYB30* were generated via *Agrobacterium tumefaciens*-mediated embryogenic callus transformation following a standardized and reproducible protocol optimized for japonica rice (cv. Nipponbare), with reference to a recently established high-efficiency rice transformation system [31].

For overexpression vector construction: The full-length coding sequences (CDS) of *OsADF7* (GenBank accession: NM_003074.8.001420040.1) and *OsMYB30* (GenBank accession: XM_015767638) were amplified from cDNA of wild-type (WT) rice seedlings using gene-specific primers listed in Table S2. The purified polymerase chain reaction (PCR) products were digested with KpnI and XbaI (for *OsADF7*) and KpnI and BamHI (for *OsMYB30*), respectively. The digested fragments were ligated into the pCAMBIA1300-221 overexpression vector (driven by the CaMV 35S promoter) using T4 DNA ligase (TaKaRa, Dalian, China), generating 35S::OsADF7 and 35S::OsMYB30 recombinant vectors. For subcellular localization, the CDS of *OsADF7* and *OsMYB30* were cloned into the pCM1205-GFP vector using XbaI and KpnI to generate GFP-fused constructs.

For CRISPR/Cas9-mediated knockout vector construction: Specific target sites of *OsADF7* and *OsMYB30* were designed using the CRISPR-GE online toolkit (http://skl.scau.edu.cn/). The high-specificity target sequence of *OsADF7* (5′-GCAAGCTCAAGTTCCTGG-3′) and *OsMYB30* (5′-CACGGCCCCGGCAACTGG-3′) were synthesized and inserted into the pYLCRISPR/Cas9Pubi-H vector using BsaI restriction enzyme, respectively (Table S2).

All recombinant vectors were confirmed by Sanger sequencing and transformed into *Agrobacterium tumefaciens* strain GV3101 using the freeze–thaw method. Mature rice seeds were dehusked, sterilized, and inoculated on N6 callus induction medium to induce embryogenic calli. After 15 days of cultivation, vigorously growing embryogenic calli were selected for Agrobacterium infection. The calli were immersed in Agrobacterium suspension (OD_600_ = 0.5) supplemented with 200 μM acetosyringone for 10 min, blotted dry, and co-cultivated on N6 co-culture medium at 25 °C in darkness for 3 days. After co-culture, the calli were washed with sterile water containing 500 mg/L cefotaxime and transferred to N6 selection medium supplemented with 50 mg/L hygromycin B and 250 mg/L cefotaxime for resistant callus screening.

Hygromycin-resistant calli were differentiated into seedlings on MS differentiation medium, and rooted seedlings were acclimatized and transplanted to soil. Transgenic lines were verified by genomic PCR and qRT-PCR with primers provided in Table S2. Homozygous T3 transgenic lines (OE: *OsADF7-OE*, *OsMYB30-OE*; KO: *Osadf7-ko*, *Osmyb30-ko*) were obtained after three successive generations of hygromycin screening and molecular identification, ensuring genetic stability for subsequent experiments.

For *OsADF7* knockout lines, Sanger sequencing verified that *ko1* carried a 12-bp deletion causing a frameshift and premature termination codon (PTC), while *ko2* harbored a 3-bp insertion disrupting the conserved ADF domain. The expression levels of *OsADF7* pre-mRNA, mature mRNA, and NMD marker genes (*OsUPF1*, *OsUPF2*, *OsUPF3*) were detected by qRT-PCR using specific primers shown in Table S2 to validate knockout efficiency and NMD pathway activation.

### 4.3. Rice Cultivation and High-Temperature Treatment

Healthy seeds of rice (*Oryza sativa* L. cv. Nipponbare) were used for all experiments. Mature seeds were dehusked and surface-sterilized in a laminar flow hood using a 1:7 (*v*/*v*) mixture of commercial sodium hypochlorite disinfectant (84 disinfectant; main component: 5.5–6.5% sodium hypochlorite) and sterile distilled water for 12 min. The sterilized seeds were rinsed 4–5 times with sterile distilled water to remove residual disinfectant, blotted dry on sterile filter paper, and inoculated on 1/2 MS solid medium. The seeds were stratified at 4 °C for 2 days to synchronize germination, then transferred to a growth chamber for cultivation.

Seedlings were grown under controlled conditions: 22 °C, 16 h light/8 h dark photoperiod, light intensity of 100 μmol·m^−2^·s^−1^, and 60% relative humidity.

#### 4.3.1. Acute Heat Stress (37 °C) for Transcriptional Analysis

Acute heat stress (37 °C) was applied to simulate transient extreme high-temperature events in paddy fields, including midday heatwaves persisting for several hours, which trigger rapid genome-wide transcriptional reprogramming as the primary early molecular response to heat stress in rice. This temperature regime was specifically employed for temporal transcriptional profiling of the OsADF gene family, *OsMYB30*, and core heat-responsive *HSP* genes, to capture the immediate signaling cascade and regulatory dynamics underlying heat acclimation.

For temporal expression analysis of OsADF family genes and *OsMYB30*, 15-day-old rice seedlings precultured at 22 °C were exposed to 37 °C for 0, 1, 3, 6, 9, and 12 h; the fifth fully expanded leaves were sampled for total RNA isolation. To quantify the induction of heat-responsive genes in transgenic lines, 7-day-old WT, *OsADF7-OE*, and *Osadf7-ko* seedlings were subjected to 37 °C heat treatment in a controlled water bath for 0 and 6 h. The 37 °C acute heat regime was selected for its ability to rapidly and robustly activate the transcriptional program of heat-related genes, as established in previous studies [1,3,7,31].

#### 4.3.2. Chronic Moderate Heat Stress (28 °C) for Phenotypic and Cytoskeletal Analysis

Chronic moderate heat stress (28 °C) simulates long-term mild high-temperature stress during the rice growing season. This temperature represents a sustained 3–6 °C increase relative to the optimal growth range of 22–25 °C for japonica rice. It serves as a physiologically relevant agronomic condition for heat acclimation that does not induce acute cellular damage. This regime was utilized for phenotypic assessment, actin cytoskeleton observation, and functional validation of the *OsMYB30*-*OsADF7* regulatory axis, as it recapitulates adaptive growth and cytoskeletal remodeling processes that underpin long-term thermotolerance in field environments.

For phenotypic quantification and actin microfilament imaging, 3-day-old seedlings were transferred to 28 °C conditions and cultivated for 4 d. The optimal growth temperature for japonica rice (cv. Nipponbare) from 22 °C to 25 °C. As widely documented in previous studies, 28 °C represents a well-established chronic moderate heat stress model that enables investigation of adaptive growth and cytoskeletal responses without triggering acute cellular injury [28,29,31].

The dual-temperature strategy establishes a mechanistic continuum: the rapid transcriptional repression of the *OsMYB30*-*OsADF7* axis under 37 °C acute stress serves as the molecular initiation of heat acclimation, and this conserved regulatory pattern is stably maintained under 28 °C chronic stress to drive persistent actin cytoskeleton remodeling and adaptive phenotypic changes, thereby linking acute molecular responses to chronic agronomic thermotolerance.

### 4.4. Quantitative Real-Time PCR (qRT-PCR)

Total RNA was extracted from plant tissues using the EasyPure Plant RNA Kit (TransGen Biotech, Beijing, China) following the manufacturer’s instructions. First-strand cDNA was synthesized from 1 μg of total RNA using the TIANScript cDNA First Strand Synthesis Kit (TransGen Biotech, Beijing, China) according to the manufacturer’s protocols. qRT-PCR was performed using the 2 × Taq Master SYBR Green Mix Kit (TransGen Biotech, Beijing, China) on a PCR detection system. The reaction system and thermal cycling procedures were conducted strictly following the kit’s manual. Relative quantification analysis was carried out using the LightCycler 480 software (Roche Diagnostics, Mannheim, Germany). *OsActin1* was amplified as a reference gene. All gene-specific primers were synthesized by Sangon Biotech Co., Ltd. (Shanghai, China). The primer sequences are listed in Table S2, and the CDS and deduced amino acid sequences of the target genes are provided in Table S3.

### 4.5. Microfilament Pharmaceutical Treatment

To dissect the regulatory function of *OsADF7* in cytoskeletal dynamics, 5-day-old OsADF7-GFP transgenic plants were subjected to specific cytoskeleton pharmacological treatments. The seedlings were immersed in solutions containing 50 nM latrunculin B (LatB, a specific microfilament depolymerizer), 1 μM phalloidin (a specific microfilament stabilizer/polymerizer), or 50 nM oryzalin (a specific microtubule depolymerizer) for 10 min in the dark to avoid fluorescence photobleaching during treatment.

For microfilament morphological observation, cotyledons were freshly excised from 7-day-old OsADF7-GFP seedlings cultivated under normal temperature (22 °C) and chronic high-temperature stress (28 °C), respectively. Confocal laser scanning microscopy was performed using a 40× oil immersion objective with an excitation wavelength of 488 nm; key parameters (laser power, gain, pinhole) were strictly unified across groups.

Microfilament quantification: Fluorescence intensity of individual microfilaments was measured using ImageJ version 1.8.0, and the full distribution was plotted as a histogram (Figure S3). K-means clustering (k = 2) was applied to objectively classify filaments into low-fluorescence (depolymerized) and high-fluorescence (polymerized) populations, eliminating arbitrary manual thresholding. Filament length was quantified by semi-automatic tracing; ≥1000 filaments were measured per genotype to ensure statistical robustness.

### 4.6. Chromatin Immunoprecipitation Technique (ChIP)

The ChIP experiment was performed using the ChIP Assay Kit (Beyotime, Shanghai, China) following the manufacturer’s protocol with minor optimizations. Fourteen-day-old WT and OsMYB30-GFP transgenic rice seedlings were cross-linked with 1% formaldehyde for 10 min at room temperature, and the reaction was terminated with 0.125 M glycine. Nuclei were isolated, and chromatin was fragmented to 200–500 bp by sonication. The sheared chromatin was divided into three fractions: (1) +antibody (Ab) group: incubated with anti-GFP antibody (Abcam, Cambridge, UK; Cat. No. ab290) at 4 °C overnight; (2) −Ab group: incubated with normal immunoglobulin G (IgG) (Beyotime, Shanghai, China; Cat. No. A7016) as a negative control; (3) input group: an aliquot of sonicated chromatin used for normalization.

For ChIP-qPCR, two *OsADF7* promoter fragments harboring the TATCC cis-elements were amplified: Pro-169bp (-169 bp relative to the TSS) and Pro-1135bp (-1135 bp relative to the TSS), with their precise positions annotated in the promoter schematic (Figure S5). Primer sequences are provided in Table S2. A validated OsMYB30-binding region in the *OsHSP25.3* promoter (GenBank: XM_015778161.2) was used as a positive control; a non-coding intergenic region without TATCC motifs served as a negative control.

qRT-PCR was performed using the same conditions as real-time PCR. Relative enrichment was calculated by the 2^^−ΔΔCt^ method: ΔCt = Ct(target) − Ct(input), and enrichment fold = 2^^−(ΔCt(+Ab)^ − ^ΔCt(−Ab))^. All experiments included three biological replicates and three technical replicates.

### 4.7. Electrophoretic Mobility Shift Assay (EMSA)

#### 4.7.1. Probe Design and Preparation

EMSA probes were designed targeting the Pro-169bp and Pro-1135bp regions of the *OsADF7* promoter, both harboring the TATCC cis-element. Biotin-labeled (hot) probes, unlabeled (cold) probes, and mutant cold probes (TATCC mutated to TAAAA to disrupt OsMYB30 binding) were synthesized by Shanghai Sangon Biotech Co., Ltd. (Shanghai, China), with biotin modification at the 5′ end of hot probes. All probe sequences are provided in Table S2.

#### 4.7.2. Recombinant Protein Purification and EMSA Assay

The OsMYB30-GST prokaryotic expression vector was transformed into *Escherichia coli* BL21 (DE3) competent cells. Protein expression was induced with 0.5 mM isopropyl β-D-thiogalactoside (IPTG) at 16 °C for 16 h. Recombinant OsMYB30-GST protein was purified using Glutathione Sepharose 4B beads (GE Healthcare, Chicago, IL, USA) and quantified with a bicinchoninic acid (BCA) Protein Assay Kit (Thermo Fisher Scientific, Waltham, MA, USA).

EMSA was performed using the LightShift Chemiluminescent EMSA Kit (Roche, Basel, Switzerland) in a 20 μL reaction system containing 2 μL 10 × binding buffer, 1 μg poly(dI-dC), 20 fmol biotin-labeled hot probe, and 0–200 ng OsMYB30-GST protein. For competitive binding assays, 100-fold or 200-fold excess unlabeled cold probe or mutant cold probe was pre-incubated prior to hot probe addition. Reactions were incubated at 25 °C for 30 min, separated on 6% native polyacrylamide gels in 0.5× Tris-borate-EDTA (TBE) buffer at 100 V for 90 min, and transferred to nylon membranes. UV cross-linking (254 nm, 120 mJ/cm^2^) was performed, and biotin-labeled probes were detected using the kit-provided chemiluminescent substrate.

### 4.8. Determination of Heat Acclimation-Related Physiological Indicators

Survival rate assay: After 4 days of 28 °C moderate high-temperature treatment, seedlings were recovered at 22 °C for 7 days. Survival rate was calculated as the percentage of surviving seedlings to total seedlings.

Electrolyte leakage (EL) assay: EL was measured using a conductivity meter (DDS-307A). Leaf samples weighting 0.1g were immersed in 20mL deionized water, shaken at 25 °C for 2h, and initial conductivity (C1) was recorded. Samples were boiled for 30min, cooled to 25 °C, and total conductivity (C2) was recorded. EL (%) = C1/C2 × 100%.

Malondialdehyde (MDA) content assay: MDA content was determined using the thiobarbituric acid (TBA) method. Absorbance was measured at 532 nm, 600 nm and 450 nm, and MDA content was calculated according to the standard formula.

Reactive oxygen species (ROS) accumulation assay: ROS accumulation was detected using 2′,7′-dichlorodihydrofluorescein diacetate (H2DCFDA) staining. Fluorescence intensity was observed under confocal laser scanning microscopy, and relative fluorescence intensity was quantified using ImageJ.

Total chlorophyll content assay: Leaf samples weighing 0.1 g were extracted with 80% acetone in dark. Absorbance was measured at 663 nm and 645 nm, and total chlorophyll content was calculated using the Arnon formula.

Maximum photochemical efficiency of PSII (Fv/Fm) assay: Fv/Fm was measured using a portable chlorophyll fluorometer (PAM-2500) after 30 min of dark adaptation.

### 4.9. Statistical Analyses

Leaf area and microfilament parameters (bundling intensity, fluorescence density, fluorescence distribution, and average length) were quantified using ImageJ software. All statistical analyses were performed with SPSS 21.0. Two-way ANOVA with Bonferroni’s post hoc test was applied for two-factor comparisons (genotype × temperature/treatment time). One-way ANOVA with Tukey’s post hoc test was used for single-factor comparisons. All experiments were performed with three biological replicates and three technical replicates. Data are shown as means ± SE. Differences were considered significant at *p* < 0.05.

## 5. Conclusions

Our findings suggest that *OsADF7* negatively regulates rice heat acclimation by inhibiting microfilament polymerization, with the NMD pathway ensuring the effective loss of function of *OsADF7* in knockout lines. These results expand the current knowledge of *ADF* gene functions in plant stress responses and provide a potential target for genetic improvement of rice heat tolerance.

## Data Availability

The original contributions presented in this study are included in the article/Supplementary Materials. Further inquiries can be directed to the corresponding authors.

## Supplementary Materials

The following supporting information can be downloaded at: https://www.mdpi.com/article/10.3390/plants15131976/s1, Table S1: Six canonical physiological indicators; Table S2: Primer sequence; Table S3: Genes and amino acid sequences; Figure S1: Mutation analysis of *OsADF7* knockout lines and the effects on mRNA expression and NMD pathway activation; Figure S2: Schematic diagram of the *OsADF7* promoter region; Figure S3: Fluorescence intensity distribution and objective clustering of rice leaf pavement cell microfilaments; Figure S4: Expression levels of OsMYB30 in WT rice seedlings under 28 °C chronic moderate heat stress for 0, 2, 4, and 6 d; Figure S5: Schematic diagram of the *OsADF7* promoter region.

## Abbreviations

The following abbreviations are used in this manuscript:

Ab	Antibody
ADFs	Actin-Depolymerizing Factors
ANOVA	Analysis of Variance
BCA	Bicinchoninic acid
BLAST	Basic Local Alignment Search Tool
ChIP	Chromatin Immunoprecipitation
ChIP-qPCR	Chromatin immunoprecipitation-quantitative polymerase chain reaction
CDSs	Cloning the full-length coding sequences
CLSM	Confocal Laser Scanning Microscopy
CRISPR/Cas9	Clustered Regularly Interspaced Short Palindromic Repeats/CRISPR-Associated Protein 9
CTAB	Cetyltrimethylammonium bromide
EMSA	Electrophoretic Mobility Shift Assay
GFP	Green Fluorescent Protein
GST	Glutathione S-Transferase
HSPs	Heat Shock Proteins
IPTG	Isopropyl β-D-Thiogalactoside
IgG	Immunoglobulin G
LatB	Latrunculin B
MEGA	Molecular Evolutionary Genetics Analysis
NJ	Neighbor-Joining
NMD	Nonsense-Mediated mRNA Decay
OE	Overexpression
PAMs	Protospacer adjacent motifs
PCR	Polymerase chain reaction
PTC	Premature Termination Codon
qRT-PCR	Quantitative Real-Time Polymerase Chain Reaction
RMSD	Root-Mean-Square Deviation
ROS	Reactive oxygen species
SE	Standard Error
TBE	Tris-borate-EDTA
TSS	Transcription Start Site
WT	Wild-type
