# Supplementary material for: The OsMYB30-OsADF7 Axis Modulates Rice Heat Acclimation Through Actin Microfilament Dynamics
Source: Plants (Basel). 2026 Jun 26;15(13):1976. doi: 10.3390/plants15131976 (PMC13364434; doi:10.3390/plants15131976)
Supplement: Supplementary file 1 [file plants-15-01976-s001.zip › plants-4313479-supplementary.pdf]

Table S1. Six canonical physiological indicators

Genotype	Treatment	Survival rate (%)	EL (%)	MDA (nmol/g FW)	ROS (relative intensity)	Total chlorophyll (mg/g FW)	Fv/Fm
WT	22°C	100	8.3±0.7	12.4±1.2	1.00±0.07	2.3±0.2	0.87±0.05
WT	28°C	77.5±2.0	14.9±1.3	21.2±1.9	2.23±0.16	1.7±0.3	0.77±0.03
Osadf7-ko	28°C	91.6±3.4**	13.8±1.2**	13.8±1.3**	1.13±0.12**	2.2±0.1**	0.83±0.03**
OsADF7-OE	28°C	52.8±4.5**	34.9±2.6**	35.6±2.5**	3.80±0.21**	1.3±0.2**	0.70±0.02**

Table S2. Primer sequence

Gene name/GenBank ID	**Primer sequence**	**Purpose**
OsADF1<br>LOC4330233	qF: GCTTCCGCTTCATCGTGTTC<br>qR: GTCGTCATAGCTCTCACCCG	For real-time PCR
OsADF2<br>LOC4334307	qF: CATCGCCTGGTCTCCATCAA<br>qR: CGGTTGCCTGGATCTCGTAG	For real-time PCR
OsADF3<br>LOC4334594	qF: ACGTGAGGAGCAAGATGGTG<br>qR: GTGTGGTCCTTGAGCACGTC	For real-time PCR
OsADF4	qF: CAGTCCAAAAGGATGCACCG<br>qR: GCGATCCCCAATCTTGTCCA	For real-time PCR
OsADF5	qF: GCCGTCTTCGACTTCGACTT<br>qR: GCTCTTATCCTCGATGCGGT	For real-time PCR
OsADF6	qF: GGTGAGGAGCAAGATGCTGT<br>qR: TGTCCATGCTCATCTCGCTG	For real-time PCR
OsADF7<br>NM_003074.8	qF: CCTGCCAACGAATGCAGATG<br>qR: TTCTCACGCGCGATGTATCA	For real-time PCR
OsADF8	qF: TTCACTGTTATGGGTGGCGG<br>qR: TCCCTCCTGTCGTCGATCTT	For real-time PCR
OsADF9	qF: GATCAAGGTGGAGAGGCTCG<br>qR: GCTCTTCTGGCAGTTCTCGT	For real-time PCR
OsADF10	qF: CGCCGTCTACGATCTGGATT<br>qR: CGTGCCGAAATTGGTTCCTC	For real-time PCR

OsADF11	qF: ACGCCATCTACGACTTCGAC<br>qR: TACAACATCTTGGCGCGGAT	For real-time PCR
OsHSP70<br>LOC4351208	qF: CGGCAGCGGACAAGAAG<br>qR: GATGCCCTCCAGCTCCTT	For real-time PCR
OsHSP90<br>LOC4342077	qF: TATTGTCCGATGGCGGTGAG<br>qR: TTGGTCATCCCAATGCCAGT	For real-time PCR
OsHSP17.8<br>LOC_Os02g48140	qF: CAGCATCTTCCCGTCCTTCC<br>qR: CTCCACCTTGACCTCCTCCT	For real-time PCR
OsHSP18.2<br>LOC4325342	qF: CAACGTGTTCGACCCCTTCT<br>qR: CGTCTCCTTCCAGTCGATGC	For real-time PCR
OsHSP18.5	qF: CAGCATCTTCCCGTCCTTCC<br>qR: GTCTCCTTCCAGTCGATCCG	For real-time PCR
OsHSP25.3	qF: GGAGCACAAGAAGGAGGAGG<br>qR: GACCTTGCTCTTGTCGCACT	For real-time PCR
OsMYB30<br>LOC4330027	qF: GAACTACTGGAACACC<br>qR: CATGTCGATGTCGGTC	For real-time PCR
OsUPF1<br>LOC4343292	qF: GCTGCTGCTGCTGTTCTTCT<br>qR: CTTGCTGCTGCTGCTGTTGT	For real-time PCR
OsUPF2	qF: GAGCAGCAGCAGCAGAAGAA<br>qR: CTTGCTGCTGCTGCTGCTCT	For real-time PCR
OsUPF3<br>LOC4335928	qF: CAGCAGCAGCAGCAGCAAA<br>qR: TTCTGCTGCTGCTGCTGCTT	For real-time PCR
OsADF7-pre-mRNA	qF: GCTGCCAACGAATGCAGATGAG<br>qR: CCGCAGATCTTCGTCGTCATC	For real-time PCR
OsActin1<br>NM_001058705	qF: GCTGCTGCTGCTGCTGTTCT<br>qR: CTTGCTGCTGCTGCTGCTCT	For real-time PCR
ChIP-pOsADF7-139bp	qF: CGTACAGCTAGAGTTC<br>qR: GTAGTCGCCAACTACG	ChIP assay to detect the degree of binding polymerization of the

		TATCC element at 139bp of the gene *OsADF7* promoter
ChIP-pOsADF7-1135bp	qF: ATATGGACATCTCGCC<br>qR: GGATGCAGAAAGCAGT	ChIP assay to detect the degree of binding polymerization of the TATCC element at 1135bp of the gene *OsADF7* promoter
EMSA-pOsADF7-139bp	GTTGGGTAGAGTATATAGTTGGATTATAT CCAGCCTTAGCTGAGCCTAAC	EMSA assay to detect the degree of binding polymerization of the TATCC element at 139bp of the gene *OsADF7* promoter
EMSA-pOsADF7-1135bp	TCAGTTTGTTAGACAGCCTTTATCCTGCC AGGCATTTGCAGTGAATTGTG	EMSA assay to detect the degree of binding polymerization of the TATCC element at 1135bp of the gene *OsADF7* promoter
Cas9-OsMYB30 MH580288	CACGGCCCCGGCAACTGG	Construction of *OsMYB30* knockout by CRISPR-CAS9 technology
Cas9-OsADF7 MH580290	GCAAGCTCAAGTTCCTGG	Construction of *OsADF7* knockout by CRISPR-CAS9 technology
OsMYB30 LOC4330027	cF: GGATCCGATCTCCATCGACCATGGTG<br>cR: GTCGACGGTGTGGATTAGAAGGTG	Full-length gene amplification
OsADF7	cF: GAATTCATGGCGAATGCAGCATC<br>cR: CACGTGTCAGTTTGCACGGCCTC	Full-length gene amplification

Table S3. Genes and Amino Acid Sequences

OsADF1 **CDS**<br>ATGTCGAATTCGGCGTCGGGAATGGCCGTGTGTGACGAATGCAAACTCAAGTTCCTGGAACT<br>TAAGGCGAAAAGGAGCTTCCGCTTCATCGTGTTCAAGATCAATGAGAAGGTCCAGCAGGTG<br>GTGGTGGACAGGTTGGGGCAGCCGGGTGAGAGCTATGACGACTTCACTGCCTGCTTACCAGC<br>AGATGAGTGCCGCTACGCGGTATTTGATTTTGACTTTGTCACTGATGAAAACTGCCAGAAGA<br>GCAAGATATTCTTCATCTCCTGGGCTCCTGATACATCAAGGGTGAGGAGCAAGATGCTGTAT<br>GCTAGCTCCAAGGATCGGTTCAAGAGGGAGCTGGACGGCATCCAGGTGGAGCTGCAGGCCA<br>CTGACCCGAGTGAGATGAGCATGGACATCGTCAAGTCGCGAGCCCTCTGA
OsADF2 **CDS**<br>ATGGCGTTCATGCGTTCCCACTCCAATGCATCCTCCGGTATGGGGGTTGCTCCTGACATCAGG<br>GACACATTCCTTGAGCTTCAGATGAAGAAAGCATTTCGCTATGTTATCTTCAAAATCGAGGA<br>AAAGCAAAAGCAAGTTGTTGTGGAGAAGACCGGGGCAACAACTGAGAGTTATGATGATTTC<br>CTGGCATCTCTCCCAGAAAATGACTGCAGATATGCCCTCTATGATTTTGACTTTGTTACTGGG<br>GAGAATGTGCAAAAGAGCAAGATTTTCTTCATCGCCTGGTCTCCATCAACATCCCGGATCCG<br>TGCTAAGATGCTGTACTCCACCTCCAAGGATCGCATCAAGCAAGAACTTGATGGATTCCACT<br>ACGAGATCCAGGCAACCGACCCAACTGAGGTAGACCTTGAGGTCCTCCGGGAGCGGGCTCA<br>TTAA
OsADF3 **CDS**<br>ATGGCGAACGCGACGTCGGGTGTGGCGGTGAGCGAGGAGTGCAAGGCGAGGTTVVAGGAGC<br>TGAGGGCGGGGCGGGCCCACAGGTTCGTGGTGTTCAAGATCGACGACGCGATGCGGCAGGT<br>GGTGGTCGACAGGGTGGGCCCACGCGACGCCGGCTTCGACGAGCTCACCGCCAGCCTCCCC<br>GCCGACGGCTGCCGCTACGCCGTGTACGACCACGACTTCACCGTCAGCGACGCCACGGCCA<br>CGGCGGCCGCCGGCGAGGGCGGCGAGGCGCCGCGCAGCAAGATCTTCTTCGTGTCGTGGTC<br>GCCGGCGGCGGCGGACGTGAGGAGCAAGATGGTGTACGCGAGCTCCAACGAAGGGTTCAA<br>GAAGGAGCTCGACGGCGTCCAGATCGACCTGCAGGCCACCGACCCCAGCGAGCTCACCCTC<br>GACGTGCTCAAGGACCACACCTCCTAA
OsADF4 **CDS**

ATGGCAAATTCATCATCTGGAGTTGCAATTCATGATGATTGCAAGCTGAAGTTCAATGAGCT<br>ACAGTCCAAAAGGATGCACCGCTTCATAACTTTCATGATGGATAACAAGGGGAAAGAGATC<br>ATTGTGGACAAGATTGGGGATCGCACAACAAGCTATGAGGATTTCACTAGCAGCCTGCCTGA<br>AGGGGACTGCCGGTTTGCAATCTATGACTTTGACTTCCTTACTGCAGAGGATGTGCCAAAGA<br>GCAGGATATTCTATATCTTATGGTCCCCAGACAATGCAAAAGTGAGGAGCAAGATGCTTTAT<br>GCTAGCTCCAACGAAAGATTCAAGAAGGAGCTGAATGGCATTCAGTTGGAAGTGCAGGCTA<br>CTGACGCCGGCGAAATCAGTCTCGATGCGCTCAAAGATCGTGTGAAATAA
***OsADF5* CDS**<br>ATGGCAATGGCTTACAAGATGGCGACGGAGGGGATGAACGTGAAGGAGGAGTGCCAGAGG<br>TGGTTCATGGAGATGAAGTGGAAGAAGGTGCACCGGTTCGTGGTGTACAAGATCGACGAGC<br>GGTCGCGCGCCGTGCTGGTGGACAAGGTGGGCGGCCCCGGCGAAGGGTACGAGGAGCTCGT<br>CGCCGCGCTGCCCACCGACGACTGCCGCTACGCCGTCTTCGACTTCGACTTCGTCACCGTCGA<br>CAACTGCCAGAAGAGCAAGATCTTCTTCATCGCCTGGTCACCGACCGCATCGAGGATAAGA<br>GCCAAGATTCTGTACGCGACGTCGAAGCAAGGGCTGAGGCGGGTGCTTGACGGGGTCCACT<br>ACGAGGTGCAAGCCACGGACTCCTCCGAGATGGGCTACGACGTCATCCGAGGCCGCGCTCA<br>GTGA
***OsADF6* CDS**<br>ATGGCGAACTCAGCGTCGGGGATGGCCGTGGGCGACGAGTGCAAGCTCAAGTTCCAGGAGC<br>TCAAGTCGAAGAGGAGCTTCCGCTTCATCACGTTCAAGATCGACGAGCGGACGCAGCAGGT<br>GGTCGTGGACAGGCTGGGCCAGCCGGGCGACACCTACGACGACTTCACCGCCTCCATGCCC<br>GCCAGCGAGTGCCGCTACGCCGTCTTCGACTTCGACTTCGTCACCGACGAGAACTGCCAGAA<br>GAGCAAGATCTTCTTCATCTCCTGGTCGCCGGACACGTCGAAGGTGAGGAGCAAGATGCTGT<br>ACGCGAGCTCCAAGGACCGGTTCAAGAGGGAGCTGGACGGGATCCAGGTGGAGCTGCAGGC<br>GACCGATCCCAGCGAGATGAGCATGGACATCGTCAAAGCGAGAGCCCTCTGA
***OsADF7* CDS**<br>ATGGCGAATGCAGCATCTGGGATGGCTGTGGACGATGAGTGCAAGCTCAAGTTCCTGGAGCT<br>GAAGGCAAAGAGGACCTACCGCTTCATCATTTACAAGATAGACGAGAAGAAGAAGATGGTT<br>GTCGTGGAGAAGGTTGGCGAGCCCGTACTGAACTACGACGATTTTGCCGCTAGCCTCCCTGC<br>CAACGAATGCAGATGCGCCATATTCGACTACGATTTCGTGACCGAGGAGAACTGCCAGAAG

AGCAAGATATTCTTCATTGCATGGTCTCCTGATACATCGCGCGTGAGAAGCAAGATGATCTA<br>CGCGAGCTCCAAGGACAGGTTCAAGAGGGAGCTCGACGGCATTCAGGTGGAGCTCCAGGCG<br>ACCGATCCAACTGAGGTTGGCCTCGACGTGATCAGAGGCCGTGCAAACTGA
***OsADF8* CDS**<br>ATGTGTTTTATGGTCAGACCGTCCATGGAGATTCTTGGATTCACTGTTATGGGTGGCGGCTCG<br>CCGGCGTGGATCGAGGTGCCGGAGAAGAGCAAGAGCGCGTTCTGGGAGCTGATGAGGAGG<br>AAGGTGCACCGGTACGTGATATTCAAGATCGACGACAGGAGGGAGGAGATCGTCGTCGAGA<br>AGACCGGCGCGCCGTGGGAGAGCTACGACGACTTCACGGCCTCGCTTCCGGCCGACGCCGT<br>CTACGATCTGGATTTCGTCAGCGATGACAACTGCAGGAAGAGCAAGATATTCTTCATCTCCT<br>GGTCCCCTTCCCTTTCCTGCATCCGAGCCAAGACCATATACGCTGTGTGGAGGAACCAATTCC<br>GGCATGAGCTTGACGGTGTTCACTTTGAGATTCAGGCCACGGACCCTGATGACATGGATTTG<br>GAAGTTCTAAGGGGCCGAGCTAATAGAACCTGA
***OsADF9* CDS**<br>ATGGCGAATTCTGCGTCAGGGCTGGCGGTGAACGACGAGTGCAAGTTCAAGTTCCAGGAGCT<br>GAAGACGAGGAGGGGGTTCAGGTTCATCGTGTTCAAGATCGACGACAAGGCCATGGAGATC<br>AAGGTGGAGAGGCTCGGGCAGACTGCCGAGGGCTACGAGGACTTCGCCGCCACCCTCCCCG<br>CCGACGAGTGCCGCTACGCCGTCTACGACCTCGACTTCGTCACCGACGAGAACTGCCAGAA<br>GAGCAAGATCTTCTTCTTCTCCTGGTCGCCTGACACGGCGAGGACAAGGAGCAAGATGCTGT<br>ACGCGAGCTCCAAGGACAGGTTCAGGAGGGAGCTGGACGGAATCCAGTGCGAGATTCAGGC<br>CACAGACCCCAGCGAGATGAGCCTCGACATCATCAGAGCCAGAGCTCACTGA
***OsADF10* CDS**<br>ATGGTGGCGGCGGCGGCGGCGGTGTTGCCATGGGGTGGCGGCGGCTCGCCGGCGTGGATCG<br>AGGTGCCGGAGAAGAGCAAGAGCGCGTTCTGGGAGCTGAAGAGGAGGAAGGTGCACCGCT<br>ACGTGATTTTCAAGATCGACGACAGGCGGGAGGAGATCGTCGTCGAGAAGACCGGCGCGCC<br>GGGGGAGAGCTACGACGACTTCACGGCGTCGCTGCCCGCCGACGACTGCCGGTACGCCGTCT<br>ACGATCTGGATTTCGTCAGCGACGACAACTGCAGGAAGAGCAAGATATTCTTCATCTCATGG<br>TCCCCTTCTGTTTCCCGCATCCGAGCCAAGACCATATACGCCGTGTCGAGGAACCAATTTCGG<br>CACGAGCTTGACGGTGTGCACTTTGAGATTCAGGCCACGGACCCTGATGACATGGATTTGGA<br>AGTTCTCAGGGGCCGTGCTAATAGAACCTGA

***OsADF11* CDS**<br>ATGGCATTCGTCAGATCACGCGCAAATGCTTCCTCTGGAATCGGTGTAGCTGCCGAGTGCAA<br>GCAGACATTTCTGGAGCTTCAGAGGAAGAAATCACACCGCTATGTCATCTTCAAGATCGACG<br>ACAAGTGCAAGGAGGTCGTCGTCGAAAAGACAGGTTCATCGACCGAGAGCTTCGACGATTT<br>CATGGACTCACTCCCTGAATCTGACTGCCGCTACGCCATCTACGACTTCGACTTCGTCACCGA<br>GGAGAACTGCCAGAAGAGCAAGATCTTCTTCGTCGCATGGTCGCCTTCGGTTTCTCGCATCCG<br>CGCCAAGATGTTGTATGCTACCTCCAAAGAACGGTTCAGGAGAGAGCTGGATGGTGTGCACT<br>ATGAGATTCAGGCAACTGATCCGTCGGAGCTGGACATTGAGCTTCTTAGAGAGCGTGCTCAT<br>TGA
***OsHSP70* CDS**<br>ATGGCGGGCAAGGGCGAGGGTCCGGCCATCGGCATCGACCTTGGCACGACCTACTCGTGCG<br>TGGGCGTTTGGCAGCACGACCGCGTGGAGATCATCGCCAACGACCAGGGCAACCGCACCAC<br>CCCCTCCTACGTCGGCTTCACCGACTCCGAGAGGCTCATCGGAGATGCTGCCAAGAACCAGG<br>TCGCCATGAACCCCATCAACACCGTCTTTGATGCCAAGCGTCTCATTGGCAGGAGGTTTAGC<br>GATGCTTCTGTTCAGAGTGACATTAAGCTCTGGCCCTTCAAGGTGATTGCTGGACCTGGTGAC<br>AAGCCTATGATTGTTGTCCAGTACAAGGGTGAGGAGAAGCAGTTTGCTGCAGAAGAGATCTC<br>CTCCATGGTCCTCATCAAGATGCGTGAGATTGCTGAGGCCTACCTTGGCACCACCATCAAGA<br>ATGCCGTTGTCACTGTTCCTGCCTACTTCAATGACTCCCAGAGGCAGGCCACCAAGGATGCT<br>GGAGTGATTGCTGGTCTCAATGTCATGCGTATCATCAACGAGCCGACTGCTGCAGCTATTGCC<br>TATGGTCTTGATAAGAAGGCCACCAGCGTTGGTGAGAAGAATGTCCTCATCTTTGACCTTGG<br>AGGTGGTACCTTTGATGTCTCCCTCCTTACCATTGAGGAGGGTATCTTTGAGGTCAAGGCCAC<br>AGCTGGTGACACCCATCTTGGTGGTGAAGATTTTGACAATCGTATGGTCAACCACTTTGTGCA<br>AGAATTCAAGAGGAAGAACAAGAAGGATATCACTGGCAACCCCAGGGCTCTCAGGAGGTTG<br>AGGACAGCTTGTGAGAGGGCAAAGAGGACCCTGTCCTCCACTGCCCAGACCACCATTGAGA<br>TCGATTCCCTGTATGAGGGCATCGACTTCTACTCAACCATCACCCGTGCCAGGTTTGAGGAGC<br>TCAACATGGATCTCTTCAGGAAGTGTATGGAGCCTGTGGAGAAGTGCCTCAGGGATGCTAAG<br>ATGGACAAGAGCTCTGTTCATGATGTTGTCCTTGTTGGTGGCTCCACTAGGATCCCGAGGGTG<br>CAGCAGCTCCTGCAGGATTTCTTCAACGGCAAGGAGCTTTGCAAGAACATCAACCCAGATGA<br>GGCTGTTGCTTATGGTGCTGCTGTCCAGGCTGCCATCTTGAGTGGTGAGGGCAACGAGAAGG

TCCAGGACCTCCTCCTGTTGGATGTTACCCCTCTCTCTCTCGGTTTGGAGACTGCTGGTGGTGT
CATGACCGTTTTGATCCCAAGGAACACCACCATTCCCACCAAGAAGGAGCAGGTCTTCTCCA
CCTACTCCGACAACCAGCCTGGTGTGCTCATCCAGGTTTATGAGGGTGAGAGGACCAGGACA
CGTGACAACAACCTGCTGGGGAAGTTTGAGCTCTCTGGAATCCCTCCTGCTCCCAGGGGTGTT
CCACAGATCACTGTTTGCTTCGACATTGATGCCAATGGTATCCTGAACGTGTCTGCTGAGGAC
AAGACCACCGGGCAGAAGAACAAGATCACCATTACCAACGACAAGGGCAGGCTTAGCAAG
GAGGAGATTGAGAAGATGGTCCAGGAGGCCGAGAAGTACAAGTCAGAGGATGAGGAGCAC
AAGAAGAAGGTGGAGTCCAAGAACGCGCTGGAGAACTACGCCTACAACATGCGCAACACC
ATCAAGGATGAGAAGATCGCCTCGAAGCTCCCGGCAGCGGACAAGAAGAAGATCGAGGAT
GCCATCGACCAGGCCATCCAGTGGCTGGACGGCAACCAGCTCGCTGAGGCTGATGAGTTCG
ATGACCGATGAAGGAGCTGGAGGGCATCTGCAACCCCATCATCGCCAAGATGTACCAGGGC
GCTGGCGCGGACATGGCCGGCGGCATGGACGAGGACGATGCTCCCCCGGCTGGCGGCAGCG
GTGCTGGCCCCAAGATCGAGGAGGTCGACTAA

OsHSP90 **CDS**

ATGGCGCCGGCGCTGAGCAGGAGCCTGGGGGCGTCGTCCGTTGCGGCGCTGAGGCCGACCC
CGTCGCGGGGCCGGGGTCCCACGCTGCGGAGCGCGGTCGCGGTGCAGGGGAGGGGAGCGGC
GGCGGTGGCCGCGAGAGGGGTGAGGTGGGAGGCCGGGAGGAGGAAGGGGAAGGGGAGGA
TGGTCGGGGTCAGGTGCGAGGCCGCCGTCACCGAGAAGCCCGCCGGGGAGGAGGAGGCGG
CCGGCGAGCAGTTCGAGTACCAGGCTGAGGTCAGCCGATTGCTGGATTTGATTGTCCACAGC
CTGTATAGCCACAAGGAGGTTTTTCTCCGTGAACTCGTAAGTAATGCAAGTGACGCGCTGGA
TAAGCTGAGATTTCTCAGTGTAACTGATTCCTCTGTATTGTCCGATGGCGGTGAGTTGGAAAT
TAGGATCAAACCCGACCCAGAGGCTGGCACAATTACTATCACTGATACTGGCATTGGGATGA
CCAAAGATGAACTCAAAGATTGCCTTGGAACCATCGCCCAAAGTGGCACCTCCAAATTTTTG
AAGGCTCTTAAGGAGAACAAAGATCTTTGGTGCAGATAATGGACTTATTGGTCAATTTGGTG
TGGGATTTTATTCGGCTTTTCTTGTTGCAGAGAAGGTTGTGGTTTCCACTAAGAGTCCAAAGG
CAGACAAACAGTATGTATGGGAAGCTATGGCTGACAGCAGCTCATATGTTATTAAGGAAGA
AACCGATCCTGAGAAAATGTTGACACGCGGAACACAGATTACTCTGTTTTTAAGAGATGATG
ATAAGTACGAGTTTGCTGACCCTGGACGTATTCAAGGTTTAGTTAAGAACTATTCCCAGTTTG
TTTCATTCCCCATATATACATGGCAGGAGAAATCAAGAACAGTTGAGGTTGAAGAAGAAGA

ACCGAAAGAAGGTGAAGAGGCAACAGAGGGTGAAAAGAAGAAGAAAAAGAAAACAATCA
CTGAGAAGTACTGGGATTGGGAATTGGCTAATGAAACAAAGCCCATATGGATGAGAAATCC
AAAGGAAGTTGAGAAAACTGAGTACAATGAATTCTACAAGAAGGCATTCAATGAGTTTTTG
GATCCTCTTGCTTACACCCACTTTACAACAGAGGGTGAGGTGGAATTCAGGAGCGTCCTCTA
CATTCCAGGAATGGCACCTCTTAGCAATGAGGAGATAATGAACCCTAAGACCAAGAATATC
CGGCTGTATGTTAAGAGAGTCTTCATATCAGATGACTTCGATGGCGAGTTGTTCCCTAGATAC
TTAAGCTTTGTAAAGGGTGTAGTGGACTCAAATGATCTTCCTCTCAATGTTTCCCGTGAGATT
CTTCAAGAAAGTCGTATTGTCAGGATCATGCGCAAAAGACTTGTCAGGAAGACATTTGATAT
GATTCAGGAGATTGCTGAGAAAGAGGACAAGGAGGACTACAAAAAATTTTGGGAGAGTTTT
GGCAAATTTGTTAAACTTGGCTGCATTGAGGACACAGGAAATCACAAACGCCTTGCTCCTCT
GTTGCGGTTTTACTCTTCCAAAAATGAGACAGATTTGATAAGTCTTGATCAGTATGTAGAGAA
CATGCCAGAAAACCAAAAGGCAATCTACTACATTGCTACAGACAGTCTTCAGAGTGCAAAG
ACTGCTCCTTTCTTGGAAAAGTTGGTTCAAAAAGACATTGAAGTTCTCTACCTTATCGAGCCG
ATTGATGAGGTTGCCATTCAGAATTTACAGACATACAAAGAGAAAAAATTTGTTGATATCAG
CAAAGAAGACCTGGAATTGGGTGATGAAGATGAGGACAAGGAAAATGAGAGCAAGCAGGA
ATACACTCTTCTATGTGACTGGATAAAGCAACAGCTTGGTGACAAAGTTGCCAAGGTTCAGA
TATCAAACCGGCTTAGCTCTTCGCCATGTGTTCTTGTATCTGGCAAATTTGGTTGGTCAGCAA
ACATGGAAAGGCTTATGAAGGCACAAACACTTGGTGATACTTCAAGCTTAGAGTTCATGAGA
GGAAGAAGAATTTTTGAAATCAACCCCGACCACCCAATTGTCAAGGACTTGAGTGCTGCTTG
CAAAAACGAGCCTGAAAGTACCGAAGCCAAGAGGGCCGTTGAGCTGTTGTACGAGACTGCG
CTGATCTCCAGTGGATATACTCCTGACAGCCCAGCTGAGTTGGGTGGCAAGATCTACGAGAT
GATGACCATCGCTCTTGGCGGGAGATGGGGAAGACCGGAGGAGTCTGAAGCCGCCACCAGC
GAATCCAACGTTGAGGTAGAGTCTTCTGAAGGTTCCGCGACGGAAGTTGTTGAGCCCTCTGA
AGTGAGGCCCGAGAGTGATCCATGGAAGGATTAA

***OsHSP17.8* CDS**

ATGTCGCTGATCCGCCGCAGCAACGTGTTCGACCCCTTCTCCCTCGACCTCTGGGACCCCTTC
GACGGCTTCCCCTTCGGCTCCGGCAGCGGCAGCATCTTCCCGTCCTTCCCGCGCGGCGCCTCC
TCCGAGACCGCGGCCGTCGCCGGCGCGCGGATCGACTGGAAGGAGACGCCCGAGGCGCACG
TGTTCAAGGCGGACGTGCCGGGGCTGAAGAAGGAGGAGGTCAAGGTGGAGGTGGAGGACG

GCAACATCCTGCAGATCAGCGGCGAGCGCAACAAGGAGCAGGAGGAGAAGACGGACCAGT GGCACCGCGTGGAGCGCAGCAGCGGCAAGTTCCTCCGCAGGTTCCGCCTCCCCGACAACGC CAAGCCGGAGCAGATCAAGGCGTCCATGGAGAACGGCGTGCTCACCGTCACCGTGCCCAAG GAGGAGGCCAAGAAGCCCGACGTCAAGTCCATCCAGATCTCCGGCTAG
***OsHSP18.2* CDS**<br>ATGTCGCTGGTGAGGCGCAGCAACGTGTTCGACCCCTTCTCCCTCGACCTCTGGGACCCCTTC GACAGCGTGTTCCGCTCCGTCGTCCCGGCCACCTCCGACAACGACACCGCCGCCTTCGCCAA CGCCCGCATCGACTGGAAGGAGACGCCGGAGTCGCACGTCTTCAAGGCCGACCTCCCCGGC GTCAAGAAGGAGGAGGTGAAGGTGGAGGTGGAGGAAGGCAACGTGCTGGTGATCAGCGGC CAGCGCAGCAAGGAGAAGGAGGACAAGAACGACAAGTGGCACCGCGTGGAGCGCAGCAG CGGGCAGTTCATGCGGCGGTTCCGGCTGCCGGAGAACGCCAAGGTGGACCAGGTGAAGGCC GGCATGGAGAACGGCGTGCTCACCGTCACCGTGCCCAAGGCCGAGGTCAAGAAGCCCGAGG TGAAGGCCATTGAGATCTCTGGCTAA
***OsHSP18.5* CDS**<br>ATGTCGCTGATCCGCCGCAGCAACGTGTTCGACCCCTTCTCCCTCGACCTCTGGGACCCCTTC GACGGCTTCCCCTTCGGCTCCGGCGGCAGCAGCAGCGGCAGCATCTTCCCGTCCTTCCCGCG CGGCGCCTCCTCCGAGACCGCGGCCTTCGCCGGCGCGCGGATCGACTGGAAGGAGACGCCC GAGGCGCACGTGTTCAAGGCGGACGTGCCGGGGCTGAAGAAGGAGGAGGTCAAGGTGGAG GTGGACGACGGCAACATCCTGCAGATCAGCGGCGAGCGCAACAAGGAGCAGGAGGAGAAG ACGGACCAGTGGCACCGCGTGGAGCGCAGCAGCGGCAAGTTCCTCCGCAGGTTCCGCCTCC CCGACAACGCCAAGCCGGAGCAGATCAAGGCGTCCATGGAGAACGGCGTGCTCACCGTCAC GGTTCCCAAGGAGGAGGCCAAGAAGCCCGACGTCAAGTCCATCCAGATCTCCGGCTAG
***OsHSP25.3* CDS**<br>ATGGCTGCTCCATTCGCTCTCGTCAGCCGTGTCTCGCCAGCGGCGCGCCTCCCCATCCGCGCC GCCTGGAGGAGGGCGAGGCCGACGGTCGGGCTCCCGTCCTCGGGGAGGGCCCGCCAGCTCG CCGTGGCCTCCGCGGCGCAGGAGAACAGGGACAACACCGCCGTCGATGTCCACGTCAACCA GGACGGCGGGAACCAGCAGGGGAACGCCGTGCAGCGCCGCCCGCGCCGCTCGTCGGCGTTG GACGGCATCTCCCCGTTCGGCCTCGTGGACCCGATGTCGCCGATGCGGACGATGCGGCAGAT GCTGGACACGATGGACCGGATTTTCGACGACGTCGCGCTGGGGTTCCCCGCCACGCCGCGGA

GGTCGCTGGCGACGGGGGAGGTGCGGATGCCGTGGGACGTCATGGAGGACGACAAGGAGGT

GAGGATGCGGTTCGACATGCCGGGCCTGTCGCGGGAGGAGGTGAAGGTGATGGTGGAGGAC

GACGCGCTCGTCATCCGCGGGGAGCACAAGAAGGAGGAGGGCGAGGGCGCGGAGGGCTCC

GGCGACGGGTGGTGGAAGGAGCGCAGCGTGAGCTCCTACGACATGCGGCTCGCGCTCCCCG

ACGAGTGCGACAAGAGCAAGGTCCGCGCCGAGCTCAAGAACGGCGTGCTGCTCGTCACCGT

GCCCAAGACGGAGGTGGAGCGCAAGGTCATCGACGTGCAGGTCCAGTAG

***OsMYB30* CDS**

ATGGTGAGGCCGCCGTGCTGCGACAAGGACGGCGTCAAGAAGGGCCCGTGGACGCCGGAG

GAGGACCTCGTCCTCGTCTCCTACGTCCAGGAGCACGGCCCCGGCAACTGGCGCGCCGTCCC

GACCAGAACAGGGCTGATGCGGTGCAGCAAGAGCTGTAGGCTCCGGTGGACCAACTACCTG

AGGCCCGGGATCAAGCGGGGAAACTTCACCGACCAGGAGGAGAAGCTCATCGTCCACCTCC

AGGCGCTCCTCGGCAACCGCTGGGCGGCCATCGCGTCGTACCTCCCCGAGCGCACGGACAA

CGACATCAAGAACTACTGGAACACCCACCTCAAGCGCAAGCTGCAGGGCGGCGGCGCCGGC

GGTGACGGAGACGGTGGCGCCGCCGGCGACGCGGTCTCCCCGAAGCCCGCCGCGCAGAGGC

CCGCGTCGTCGTCCAAGGGGCAGTGGGAGCGGCGCCTGCAGACCGACATCGACATGGCGCG

CCGCGCGCTCCGCGAGGCGCTCACGTCGCTCGACGACGTCAAGCCACCGCATCAGCCCGAC

GCCGCCAACGCCGCCGCGGGGGGAGGCGGCGCCACCACCGGAGCAGCAGCGTCCGCCGGC

GCCGATAGCCCCGCCGCGTCGAGCACGTCGGGGGCGTCGCAGTGCTCCCCGTCATCCGCCGG

CTACGTCCTCACCACCGAGAACATCTCCCGGATGCTCGACGGGTGGGCCCGGAAGAAGGGC

GGCGGCGGCGGCAGGCGCGCCGCCGGCAGCGGGCCGGCCACCCCCGGCGCCACCGAGAGC

GCGTCCGGCTCGTCGGAAGCCTCCGAGGTGTCCTACGGCGGCACGGCCTTGTCCGCAGCGGC

GGCGCCCGCGTCCGCCTTCGAGTACGAGACAAAGCCGACCGTCACCGCCGCCGCCGCCGCC

GACGCCGGCGACGAGACGCAGCTCTCCGCCATCGAGTCGTGGCTGTTCGCCGACGCCGACG

GCATCGAGAGTGGCAGCTTGCTCGACGCGGCCATGGATTACACCTTCTAA

OsMYB30 Amino acids

MVRPPCCDKDGVKKGP*WT*PEEDLVLVSYVQEHGPGNWRAVPTRTGLMRCSKSCRLR*WT*NYLRP

GIKRGNFTDQEEKLIVHLQALLGNRWAAIASYLPERTDNDIKNYWNTHLKRKLQGGGAGGDGDG

GAAGDAVSPKPAAQRPASSSKGQWERRLQTDIDMARRALREALTSLDDVKPPHQPDAANAAAG

GGGATTGAAASAGADSPAASSTSGASQCSPSSAGYVLTTENISRMLDGWARKKGGGGGRRAAGS

GPATPGATESASGSSEASEVSYGGTALSAAAAPASAFEYETKPTVTAAAAADAGDETQLSAIESWL<br><br>FADADGIESGSLLDAAMDYTF

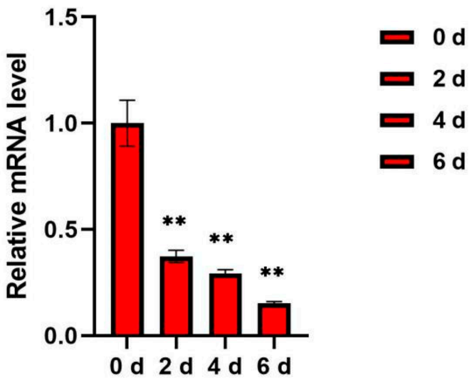

Figure S1. Expression levels of *OsADF7* in WT rice seedlings under 28°C chronic moderate heat stress for 0, 2, 4, and 6 d. Data are means ± SE, n = 3. ** *P < 0.01* vs. 0 d.

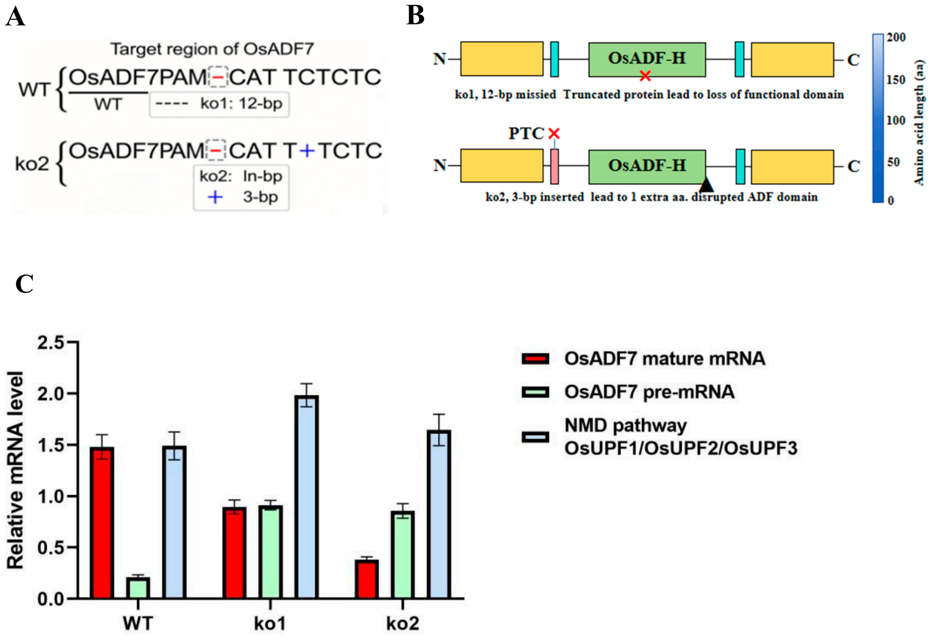

Figure S2. Mutation analysis of *OsADF7* knockout lines and the effects on mRNA expression and NMD pathway activation. (A) Sanger sequencing confirms *OsADF7* target region mutations: a 13-bp deletion in ko1 and a 3-bp insertion in *ko2*, altering the sequence to "CATT + TCTC". Schematic of *OsADF7* domain disruption: the 12-bp deletion in ko1 causes a frameshift mutation, introducing a premature termination codon (PTC) that truncates the protein and eliminates the functional ADF domain; the 3-bp insertion in ko2 adds one amino acid, disrupting the conserved ADF domain. The right scale indicates resulting protein amino acid lengths. Relative expression analysis: mature *OsADF7* mRNA is significantly reduced in

ko1 and *ko2*, while pre-mRNA levels remain unchanged, indicating unaltered transcription initiation. NMD pathway markers (*OsUPF1*, *OsUPF2*, *OsUPF3*) are upregulated, confirming NMD-mediated degradation of aberrant transcripts.

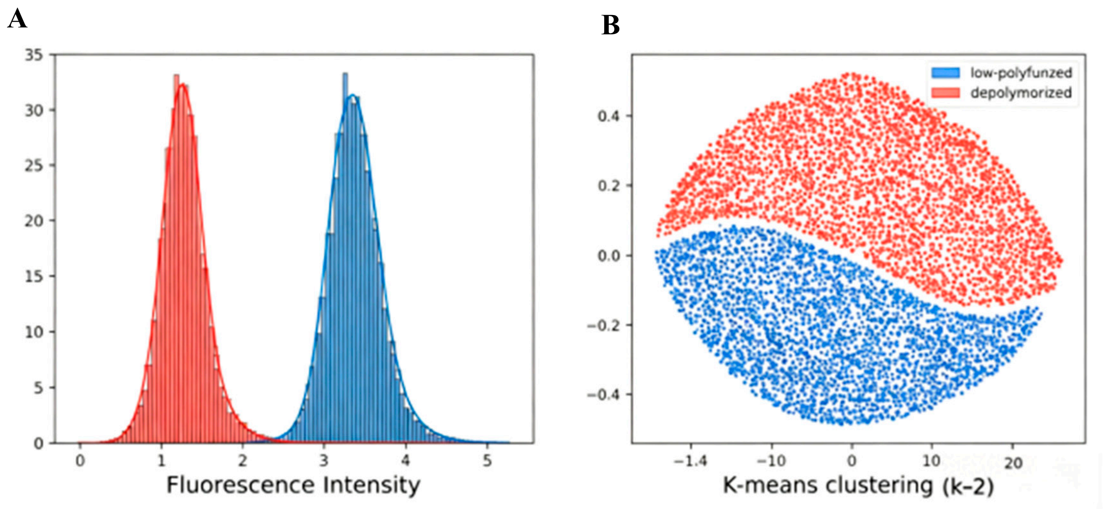

Figure S3. Fluorescence intensity distribution and objective clustering of rice leaf pavement cell microfilaments.

(A) Histogram of full-scale microfilament fluorescence intensity showing a typical bimodal distribution.

(B) K-means clustering (k=2) result objectively dividing microfilaments into low-fluorescence (depolymerized) and high-fluorescence (polymerized) populations.

Note: This clustering replaces the arbitrary 0-20/20-100 threshold and validates the robustness of microfilament classification.

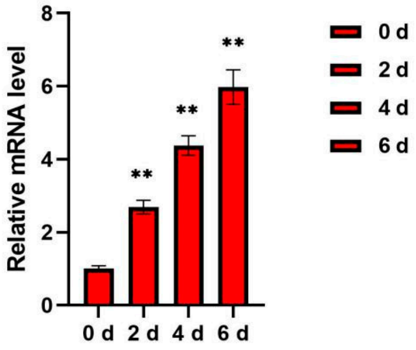

Figure S4. Expression levels of *OsMYB30* in WT rice seedlings under 28°C chronic moderate heat stress for 0, 2, 4, and 6 d. Data are means ± SE, n = 3. ** *$P < 0.01$* vs. 0 d.

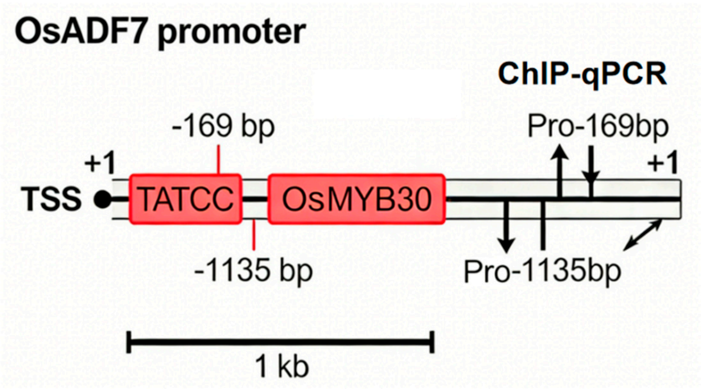

Figure S5. Schematic diagram of the *OsADF7* promoter region. The transcription start site (TSS) is marked as +1. Note: The positions of the TATCC *cis*-elements (binding sites for OsMYB30) at -169 bp and -1135 bp upstream of TSS are indicated by red boxes. The black arrows represent the ChIP-qPCR primers for the Pro-169bp and Pro-1135bp regions. The scale bar indicates the length of the promoter region (1 kb).
